# Bicyclic Pyrrolidine-Isoxazoline γ Amino Acid: A Constrained Scaffold for Stabilizing α-Turn Conformation in Isolated Peptides

**DOI:** 10.3389/fchem.2019.00133

**Published:** 2019-03-18

**Authors:** Francesco Oliva, Raffaella Bucci, Lucia Tamborini, Stefano Pieraccini, Andrea Pinto, Sara Pellegrino

**Affiliations:** ^1^Department of Chemistry, University of Milan, Milan, Italy; ^2^Department of Pharmaceutical Sciences, University of Milan, Milan, Italy; ^3^Department of Food, Environmental and Nutritional Sciences, University of Milan, Milan, Italy

**Keywords:** unnatural γ-amino acids, peptidomimetic, isoxazoline, α-turn, metadynamic studies, conformational analysis

## Abstract

Unnatural amino acids have tremendously expanded the folding possibilities of peptides and peptide mimics. While α,α-disubstituted and β-amino acids are widely studied, γ-derivatives have been less exploited. Here we report the conformational study on the bicyclic unnatural γ amino acid, 4,5,6,6a-tetrahydro-3a*H*-pyrrolo[3,4-d]isoxazole-3-carboxylic acid **1**. In model peptides, the (+)-(3a*R*6a*S*)-enantiomer is able to stabilize α-turn conformation when associated to glycine, as showed by ^1^H-NMR, FT-IR, and circular dichroism experiments, and molecular modeling studies. α-turn is a structural motif occurring in many biologically active protein sites, although its stabilization on isolated peptides is quite uncommon. Our results make the unnatural γ-amino acid **1** of particular interest for the development of bioactive peptidomimetics.

## Introduction

Amino acids are the key building blocks of proteins and biomolecules and are widely exploited in different applications, from pharmaceutical chemistry and biomedicine to material science, optoelectronics and catalysis (Zhang et al., 2012; Mikhalevich et al., 2017; Solomon et al., 2017; López-Andarias et al., 2018; Raymond and Nilsson, 2018). The insertion of unnatural amino acids (UAAs) in peptides and peptide mimics could add specific features to these molecules, such as proteolytic stability, active functional groups and new reactivity (Clerici et al., 2016; Pellegrino et al., 2016, 2017; Bucci et al., 2017). α,α disubstituted and β- homologs of natural amino acids have been particularly studied during the years for their ability to introduce conformational constrains in peptides and to stabilize specific secondary structures (Bonetti et al., 2015; Pellegrino et al., 2015; Fanelli et al., 2017; Kobayashi et al., 2017). On the other hand, γ UAAs provide a further opportunity to engineer the available backbone through the incorporation of an additional methylene group (Vasudev et al., 2011; Sohora et al., 2018). Recent studies report that γ-peptides are able to form helices, sheets and turns, whose conformational stability is increased by introducing substituents on the backbone chain (Bouillère et al., 2011). Moreover, cyclic γ UAAs, as gabapentin (Gpn) (Chatterjee et al., 2009; Konda et al., 2018) and γ-cyclohexyl amino acid (Guo et al., 2012) are able to stabilize both turn and helix conformation in oligomers and in mixed α-γ and β-γ sequences. On the other hand, the use of bicyclic γ amino acids for the stabilization of the peptide structure is much less investigated (Machetti et al., 2000).

In this work, we investigated the conformational behavior of both the enantiomers of the bicyclic unnatural γ amino acid 4,5,6,6a-tetrahydro-3a*H*-pyrrolo[3,4-*d*]isoxazole-3-carboxylic acid **1**, obtained starting from the corresponding ethyl esters recently described by us (Tamborini et al., 2015). Compound **1** is a conformationally constrained dipeptide analog and, in principle, it could substitute two amino acids in a peptide chain. The presence of the constrained bicyclic system could induce specific secondary structure allowing a proper orientation of the peptide arms at C- and N- termini. Furthermore, the presence of the isoxazoline ring, a core often found in biologically active compounds, could be particularly useful for future applications in the pharmaceutical field. In fact, isoxazoline derivatives are important scaffolds found in many naturally occurring and biologically active compounds possessing a wide range of bioactivities, such as antibacterial, antifungal, antiparasitic (Conti et al., 2011; Bruno et al., 2014; Pinto et al., 2016a), anticancer (Castellano et al., 2011; Kaur et al., 2014), anti-inflammatory and anticonvulsant activity (Sperry and Wright, 2005; Pinto et al., 2011, 2016b). Isoxazolines are also considered to be important precursors for the synthesis of β-hydroxyketones (Kozikowski and Park, 1990; Tsantali et al., 2007), β-aminoalcohols (Fuller et al., 2005), isoxazolidines (Itoh et al., 2002), and many other valuable compounds. Recently, peptidomimetics containing the isoxazoline ring have been reported as β-turn mimics (Bucci et al., 2018; Memeo et al., 2018).

**Figure 1:**
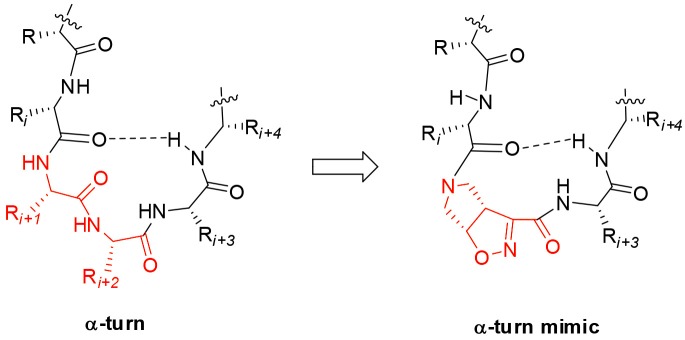
Natural α-turn and the proposed α-turn mimic containing the bicyclic pyrrolidine-isoxazoline γ amino acid.

Starting from the (–)-(3a*S*6a*R*)-**1** and (+)-(3a*R*6a*S*)-**1** enantiomers, model peptides containing Leu-Val dipeptide at *C*-terminus and variable sequences at *N*-terminus were prepared. Their conformational behavior was investigated by NMR spectroscopy, FT-IR, circular dichroism, and molecular modeling. Our results indicated that (+)-(3a*R*6a*S*)-**1** enantiomer, in combination with glycine, is effective in stabilizing the α-turn conformation in peptides (Figure 1). This structural motif occurs quite often in many key sites of proteins, such as enzyme active site, and metal binding domains (Wintjens et al., 1996), although few molecules are known to mimic or stabilize it on isolated peptides (Kelso et al., 2004; Hoang et al., 2011; Krishna et al., 2014; Wang et al., 2018.). Our results make thus the unnatural γ-amino acid **1** of particular interest for future development of bioactive peptidomimetics.

## Materials and Methods

### Materials

Chemicals were obtained from Zentek (Italy) and used without further purification. Melting points were determined in a Stuart Scientific melting point apparatus in open capillary tubes and are certified. ESI mass spectra were recorded on an LCQESI. MS was recorded on a LCQ Advantage spectrometer from Thermo Finningan and a LCQ Fleet spectrometer from Thermo Scientific. CD experiments were carried out on a Jasco J-810 instrument. Spectra were obtained from 195 to 250 nm with a 0.1 nm step and 1 s collection time per step, taking three averages. The CD spectra were plotted as mean residue ellipticity θ (degree × cm^2^ × dmol^−1^) vs. wave length λ (nm). Noise-reduction was obtained using a Fourier-transform filter program from Jasco. The NMR spectroscopic experiments were carried out on a Varian OXFORD 300 MHz (300 and 75 MHz for ^1^H and ^13^C, respectively). Chemical shifts, δ, are given in ppm relative to the CHCl_3_ internal standard, and the coupling constants, *J*, are reported in hertz (Hz).

### Methods

#### Synthesis of (–)-(3a*S*,6a*R*)-1 and (+)-(3a*R*,6a*S*)-1

Compound (**–**)-**2** or (+)-**2** (200 mg, 0.70 mmol) was dissolved in MeOH (3.0 mL) and treated with 1N NaOH aqueous solution (1.4 mL). The mixture was stirred at room temperature for 1 h and the disappearance of the starting material was monitored by TLC (Cyclohexane/EtOAc 7:3). The mixture was diluted in water (20 mL), made acidic with 2N aqueous HCl and extracted with EtOAc. The organic phase was dried over anhydrous Na_2_SO_4_ and after evaporation of the solvent, the acid derivate (–)-(3a*S*,6a*R*)**-1** or (+)-(3a*R*,6a*S*)**-1** (170 mg, 95%) was obtained as a white solid.

**(–)-(3a*S*,6a*R*)-1:** R_*f*_ (CH_2_Cl_2_/MeOH 9:1 + 1% AcOH): 0.40; [α]${}_{\text{D}}^{20}$: (–) 192.2 (c: 0.55 in MeOH); mp = dec. *T* > 137°C; ^1^H NMR (300 MHz, CD_3_OD): δ 5.35 (dd, *J* = 4.8, 9.2, 1H); 4.10 (ddd, *J* = 2.2, 9.2, 9.2, 1H); 3.86–3.96 (m, 2H); 3.38–3.50 (m, 2H); 1.44 (s, 9H) ppm; ^13^C NMR (75 MHz, CD_3_OD): δ 161.28, 154.77, 152.95, 87.62, 80.33, 53.06, 50.93, 49.10, 27.12 (3C) ppm.

**HRMS** (ESI) [M + Na]^+^ calculated for C_11_H_16_N_2_O_5_Na: 279.0957, found: 279.0950.

**(+)-(3a*R*,6a*S*)-1:** [α]${}_{\text{D}}^{20}$: (+) 192.5 (c: 0.57 in MeOH).

### General Procedure for Coupling Reaction

Free carboxylic acid (1 equiv.) was dissolved in CH_2_Cl_2_ (0.01M solution) and the mixture was cooled to 0°C. HOBt (1.1 equiv.) and EDC (1.1 equiv.) were added. After 1 h, free amino compound (1 equiv.) was added, followed by the addition of DIEA (2 equiv.). The reaction mixture was stirred at room temperature for 24 overnight (TLC analysis). The organic layer was washed with a 5% solution of KHSO_4_ (3 times), with a saturated solution of NaHCO_3_ (3 times) and with brine (1 time). The organic phase was dried over Na_2_SO_4_ and the solvent was removed under vacuum. The products were purified by column flash chromatography on silica gel (hexane/ethyl acetate gradient) followed by a crystallization from a mixture of ethyl acetate/hexane.

**Table:** 

**Product**	**Yield (%)**	**Product**	**Yield (%)**
*N*-Boc-Leu-Val-CONH${}_{2}^{\text{a}}$	96	**8**	77
**4**	65	**9**	80
**5**	63	**10**	65

a *see Steinke and Kula (1990)*.

#### N-Boc-(–)-Δ^2^-isox-Leu-Val-CONH_2_ (4)

^1^H NMR (300 MHz, CDCl_3_): δ 7.11 (bs, 1H), 6.88 (d, *J* = 8.8 Hz, 1H), 6.19 (bs, 1H), 5.72 (bs, 1H), 5.34–5.29 (m, 1H), 4.56–4.48 (m, 1H), 4.31 (dd, *J* = 8.2; 6.9 Hz, 1H), 4.17–3.83 (m, 3H), 3.56 – 3.42 (m, 2H), 2.23–2.07 (m, 1H), 1.85–1.61(m, 3H), 1.43 (s, 9H), 0.99 – 0.92 (m, 12H) ppm.

^13^C NMR (75 MHz, CDCl_3_): δ 173.04, 171.32, 159.44, 154.28, 153.93, 87.40, 80.43, 58.24, 53.26, 52.30, 50.53, 49.32, 40.87, 30.53, 28.31, 24.82, 22.91, 21.92, 19.27, 17.93 ppm.

HRMS (ESI) [M + Na]^+^ calculated for C_22_H_37_N_5_O_6_Na: 490.2642, found: 490.2635.

#### N-Boc- (+)-Δ^2^-isox-Leu-Val-CONH_2_ (5)

^1^H NMR (300 MHz, CDCl_3_): δ 7.05 (bs, 1H), 6.73 (d, *J* = 7.9 Hz, 1H), 6.13 (bs, 1H), 5.69 (bs, 1H), 5.37–5.28 (m, 1H), 4.56–4.47 (m, 1H), 4.36–4.27 (m, 1H), 4.18–4.07 (m, 1H), 3.99–3.77 (m, 2H), 3.61–3.41 (m, 2H), 2.28–2.10 (m, 1H), 1.78–1.57(m, 3H), 1.49 (s, 9H), 1.01 – 0.90 (m, 12H) ppm.

^13^C NMR (75 MHz, CDCl_3_): δ 173.16, 171.54, 159.33, 154.16, 153.96, 80.34, 60.38, 58.16, 53.27, 52.25, 49.41, 41.02, 30.67, 28.32, 24.83, 22.87, 21.99, 19.20, 18.02 ppm.

HRMS (ESI) [M + Na]^+^ calculated for C_22_H_37_N_5_O_6_Na: 490.2642, found: 490.2635.

#### NH-Boc-Phe-(-)-Δ^2^-isox-Leu-Val-CONH_2_ (8) –Mixture of two conformers-

^1^H NMR (300 MHz, CDCl_3_): δ 7.36–7.16 (m, 5H), 7.15–7.08 (m, 1H), 7.05–6.97 (m, 1H), 6.88–6.77 (m, 1H), 6.05 (bs, 1H), 5.71 (bs, 1H), 5.43 (d, *J* = 8.4 Hz 1H), 5.33–5.22 (m, 3H), 5.09–4.9 (m, 1H), 4.64–4.44 (m, 3H), 4.33–4.23 (m, 2H), 4.17–4.03 (m, 2H), 3.99–3.91 (m, 1H), 3.87–3.75(m, 2H), 3.70–3.50 (m, 2H), 3.42–3.30 (m, 1H), 3.13–3.03 (m, 1H), 2.97–2.74 (m, 3H), 2.51(dd, *J* = 12.2; 4.8 Hz, 1H), 2.21–2.05(m, 1H), 1.51–1.33 (m, 9H), 1.06 – 0.84 (m, 12H) ppm.

^13^C NMR (75 MHz, CDCl_3_): δ 172.91, 171.38, 170.09, 159.11, 157.88, 157.30, 153.83, 152.33, 136.34, 129.46, 129.30, 128.56, 128.44, 127.12, 126.85, 96.33, 87.21, 85.98, 58.09, 53.31, 52.77, 52.36, 50.95, 49.64, 49.00, 48.59, 41.05, 40.91, 30.83, 30.62, 29.69, 28.35, 24.84, 22.81, 22.71, 19.18, 17.94 ppm.

HRMS (ESI) [M + Na]^+^ calculated for C_31_H_46_N_6_O_7_Na: 637.3326, found: 637.3332.

#### NH-Boc-Phe-(+)-Δ^2^-isox-Leu-Val-CONH_2_ (9) –Mixture of two conformers-

^1^H NMR (300 MHz, CDCl_3_): δ 7.33–7.05 (m, 6H) 7.04–6.91 (m, 1H), 6.70 (d, *J* = 8.5 Hz, 1H), 6.13–5.91 (m, 2H), 5.46 (d, *J* = 8.5 Hz, 1H), 5.36–5.10 (m, 2H), 4.87–4.71 (m, 1H), 4.67–4.43 (m, 1H), 4.38–4.23 (m, 2H), 4.23–3.69 (m, 4H), 3.69–3.41 (m, 1H), 3.21 (dd, *J* = 14.3, *J* = 5.0 Hz, 1H), 3.10–2.79 (m, 2H), 2.54 (dd, *J* = 14.3, *J* = 5.0 Hz, 1H), 2.30–1.99 (m, 1H), 1.89–1.55 (m, 3H), 1.49 (m, 9H), 1.31 (m, 12 H) ppm.

^13^C NMR (75 MHz, CDCl_3_): δ 173.14, 172.90, 171.92, 171.23, 170.66, 170.49, 159.68, 158.86, 155.92, 155.18, 153.77. 153.59, 136.00, 129.63, 129.43, 129.15, 128.60, 128.41, 127.14, 127.05, 86.96, 85.53, 79.99, 79.88, 58.23, 57.68, 54.13, 53.47, 52.97, 52.19, 51.05, 48.82, 41.04, 40.85, 39.32, 30.88, 30.66, 28.45, 28.30, 25.05, 24.81, 22.91, 22.01, 21.51, 19.28, 17.98, 17.84 ppm.

HRMS (ESI) [M + Na]^+^ calculated for C_31_H_46_N_6_O_7_Na: 637.3326, found: 637.3332.

#### NH-Boc-Phe-Gly-(+)-Δ^2^-isox-Leu-Val-CONH_2_ (10)

^1^H NMR (300 MHz, CD_3_CN): δ 7.47 (d, *J* = 6.9 Hz, 1H), 7.30–7.26 (m, 6H), 6.95 (d, *J* = 8.5 Hz, 1H), 6.72 (bs, 1H), 6.42 (bs, 1H), 5.63 (bs, 1H), 5.47–5.31 (m, 1H), 4.52–4.47 (m, 1H), 4.40–3.39 (m, 8H), 3.23–3.07 (m, 1H), 2.70–2.93 (m, 1H), 2.14–2.08 (m, 1H), 1.78–1.55 (m, 3H), 1.35 (s, 9H), 1.02–0.77 (m, 12H) ppm.

^13^C NMR (75 MHz, CD_3_CN): δ 173.43, 171.63, 167.09, 159.52, 154.34, 137.64, 129.27, 128.31, 126.53, 109.99, 87.41, 86.02, 79.09, 58.06, 56.82, 55.76, 53.67, 52.65, 51.39, 49.79, 48.71, 41.62, 40.43, 40.10, 37.60, 31.51, 30.55, 29.34, 27.49, 24.77, 22.27, 20.81, 18.86, 17.21, 16.63 ppm.

HRMS (ESI) [M + Na]^+^ calculated for C_33_H_49_N_7_O_8_Na: 694.3540, found: 694.3539.

### General Procedure for *N*-Boc-Deprotection

*N*-Boc protected compound (1 equiv.) was dissolved in CH_2_Cl_2_ (0.01M) and the solution cooled at 0°C. TFA (50% v/v) was added dropwise, the solution was warmed up at room temperature and was stirred for 2 h. The solvent was removed under vacuum with the obtainment of the trifluoroacetate salt that was directly used in the next coupling step.

**Table:** 

**Product**	**Yield (%)**
**3**^a^	95
**6**	95
**7**	95

a *see Smith and Spackman (1955)*.

#### NH-(–)-Δ^2^-isox-Leu-Val-CONH2 (6)

^1^H NMR (300 MHz, CD_3_OD): δ 7.90 (d, *J* = 8.7 Hz, 1H), 5.53 (dd, *J* = 9.5, 4.9 Hz, 1H), 4.54 (dd, *J* = 9.8, 4.9 Hz, 1H), 4.42 (t, *J* = 8.8 Hz, 1H), 4.26 – 4.17 (m, 1H), 3.90 – 3.84 (m, 1H), 3.89 – 3.75 (m, 2H), 3.58 – 3.49 (m, 2H), 3.22 (dd, *J* = 5.9, 4.1 Hz, 1H), 2.05 (dq, *J* = 13.6, 6.8 Hz, 1H), 1.80 – 1.59 (m, 3H), 1.05 – 0.82 (m, 12H) ppm.

^13^C NMR (75 MHz, CD_3_OD): δ 147.47, 172.82, 159.52, 152.51, 85.48, 58.22, 52.96, 52.21, 50.55, 49.27, 40.31, 30.68, 24.54, 21.98, 20.47, 18.32, 17.08 ppm.

HRMS (ESI) [M + Na]^+^ calculated for C_17_H_29_N_5_O_4_Na: 390.2117, found: 390.2111.

#### NH-(+)-Δ^2^-isox-Leu-Val-CONH2 (7)

^1^H NMR (300 MHz, CD_3_OD); δ 7.97 (d, *J* = 8.6 Hz, 1H), 5.53 (dd, *J* = 9.5, 4.9 Hz, 1H), 4.55 (m, 1H), 4.39 (m, 1H), 4.26 – 4.16 (m, 1H), 3.90 – 3.72 (m, 2H), 3.58 – 3.43 (m, 2H), 3.24 – 3.16 (m, 1H), 2.05 (m, 1H), 1.75 – 1.58 (m, 3H), 1.02 – 0.81 (m, 12H) ppm.

^13^C NMR (75 MHz, CD_3_OD): δ 147.47, 172.99, 159.40, 152.40, 128.72, 85.21, 58.05, 53.12, 52.17, 50.66, 49.14, 40.21, 30.91, 29.36, 24.84, 21.99, 20.25, 18.26, 16.90 ppm.

HRMS (ESI) [M + Na]^+^ calculated for C_17_H_29_N_5_O_4_Na: 390.2117, found: 390.2111.

### Molecular Modeling

Compound **9** and **10** were modeled in explicit solvent with periodic boundary conditions using metadynamics and restrained molecular dynamics simulations. For each of the compounds two isomers (*cis* and *trans* with respect to the amide bond) were considered, resulting in a set of four peptides. Such molecules were solvated in a cubic box of 4 nm with chloroform (compound **8**) and acetonitrile (compound **10**) to reproduce the NMR experiment conditions. Every system has been submitted to geometry optimization with the steepest descent algorithm with a convergence of 100 kJ mol^−1^ nm^−1^. Then we performed a 1 ns equilibration in NVT conditions at 300 K, followed by a 1 ns NPT equilibration at the 1 bar and at the same temperature. After the equilibration phase, we ran a 50 ns Well Tempered Metadynamics (WTMD) with a 8.0 kJ mol^−1^ bias-factor, at a reference temperature of 300 K. To study the open and closed state of both peptides, we selected as collective variable (CV) the distance between C_β_VAL and the *N*-*tert*-butoxy carbonyl (*N*-Boc) quaternary carbon. A Gaussian hill with σ = 0.1 Å and an initial height of 1 kJ mol^−1^ was added once every 100 steps. The simulation was sped up by saving the Gaussian hills on a grid that ranged from 0 to 16 Å and spaced 0.02 Å. Compound **10** was also simulated using a 50 ns restrained molecular dynamics. Special potentials are used for imposing restraints on the motion of the system, to include knowledge from experimental data such as the NMR derived interatomic distances. Distance restraints add a penalty to the potential when the distance between specified pairs of atoms exceeds a threshold value (Abraham et al., 2014). The threshold values used in our simulation are reported in the Supplementary Materials.

The restraints are applied in a progressive way to give time to the peptide to relax, using a τ equal to 500 ps. Since we are using multiple distance restraints, they are not necessary satisfied at each simulation step but as a time average. The atoms restrained and the relative distances are shown in Table S1. Compound **9** and **10** have been described using the Amber99SB-ILDN Force Field (Hornak et al., 2006). 4,5,6,6a-Tetrahydro-3aH-pyrrolo[3,4-*d*]isoxazole-3-carboxylic acid residues were parameterized according to standard procedures as explained in reference (Gandini et al., 2018). During molecular dynamics (MD) and metadynamics simulations, temperature was held constant with the v-rescale algorithm (Bussi et al., 2007), while pressure was kept constant through the Berendsen barostat (Berendsen et al., 1984). MD simulations were performed using the leap-frog algorithm with 2 fs time-step, with holonomic constraints on every bond enforced using the LINCS algorithm. Simulations and subsequent analysis were performed with the GROMACS 5.0.4 (Van Der Spoel et al., 2005) program suite, while metadynamics was run along with PLUMED version 2.2.2 (Tribello et al., 2014).

## Results and Discussion

**Scheme 1:**
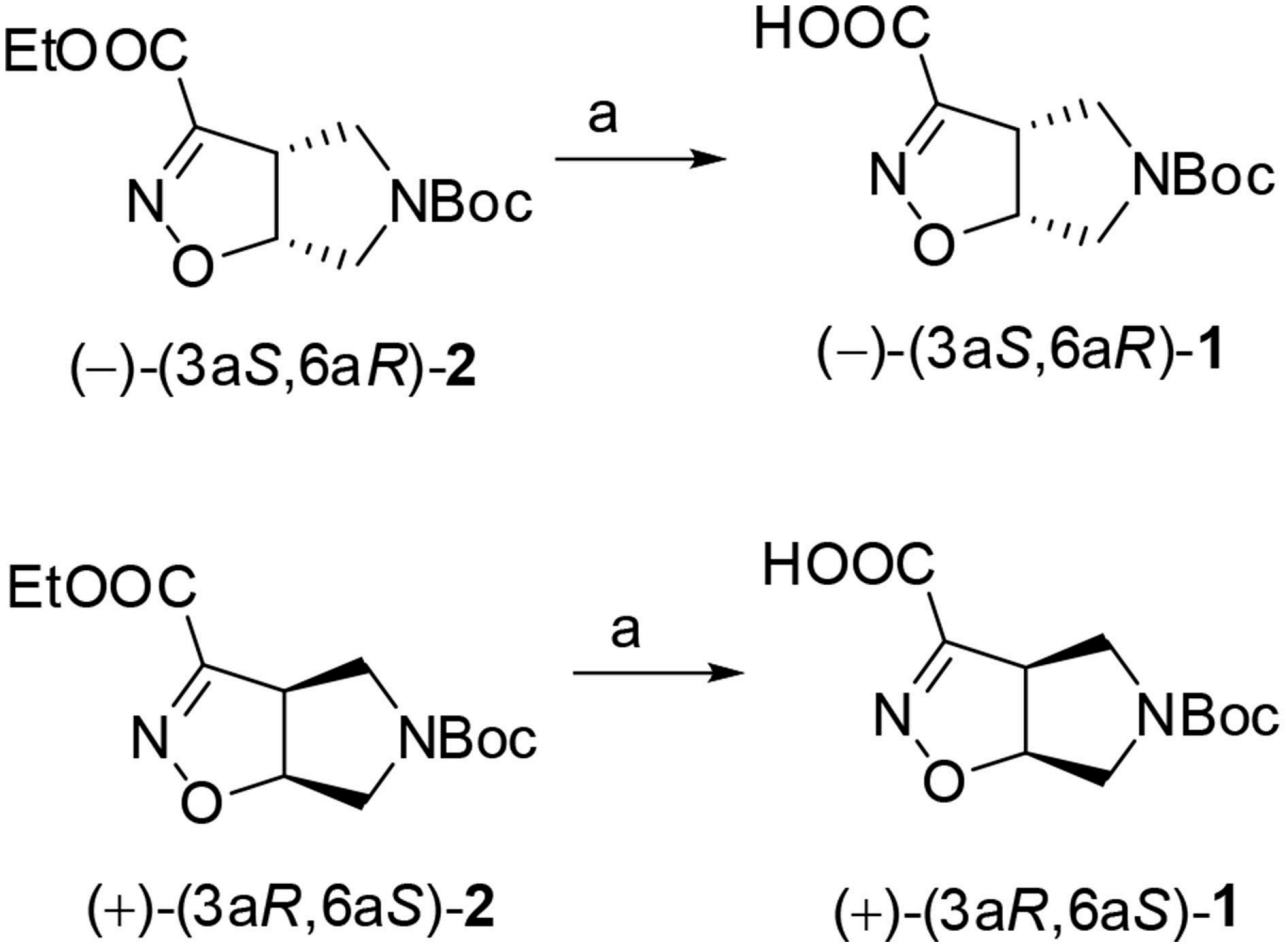
1N NaOH, MeOH, r.t., 1h.

The two enantiomers of compound **1** were obtained starting from the corresponding ethyl esters recently described by us (Tamborini et al., 2015). Racemate (±)-**2** (Conti et al., 2006) was synthesized in a flow chemistry reactor exploiting the 1,3-dipolar cycloaddition reaction of *N*-Boc-3-pyrroline with ethoxycarbonyl formonitrile oxide. An excellent enantiomeric separation (e.e. >99%) of the racemate was achieved by semi-preparative chiral HPLC. Alkaline hydrolysis of (–)-(3a*S*6a*R*)-**2** and (+)-(3a*R*6a*S*)-**2** gave the desired (–)-(3a*S*6a*R*)-**1** and (+)-(3a*R*6a*S*)-**1**, respectively (Scheme 1).

**Scheme 2:**
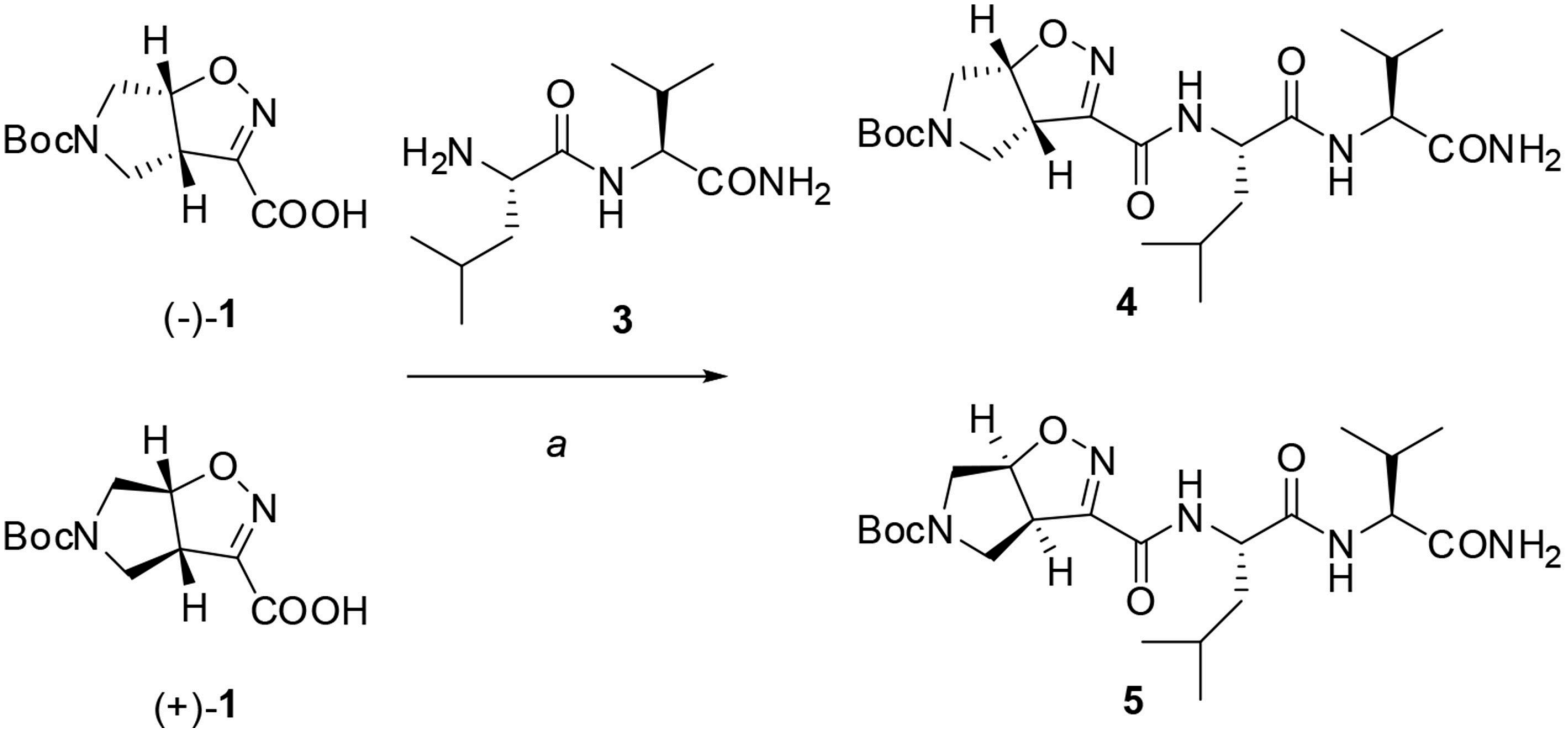
Reagents and conditions: a) EDC/HOBt/DIEA, CH_2_Cl_2_ from 0°C to r.t., 24 h.

**Scheme 3:**
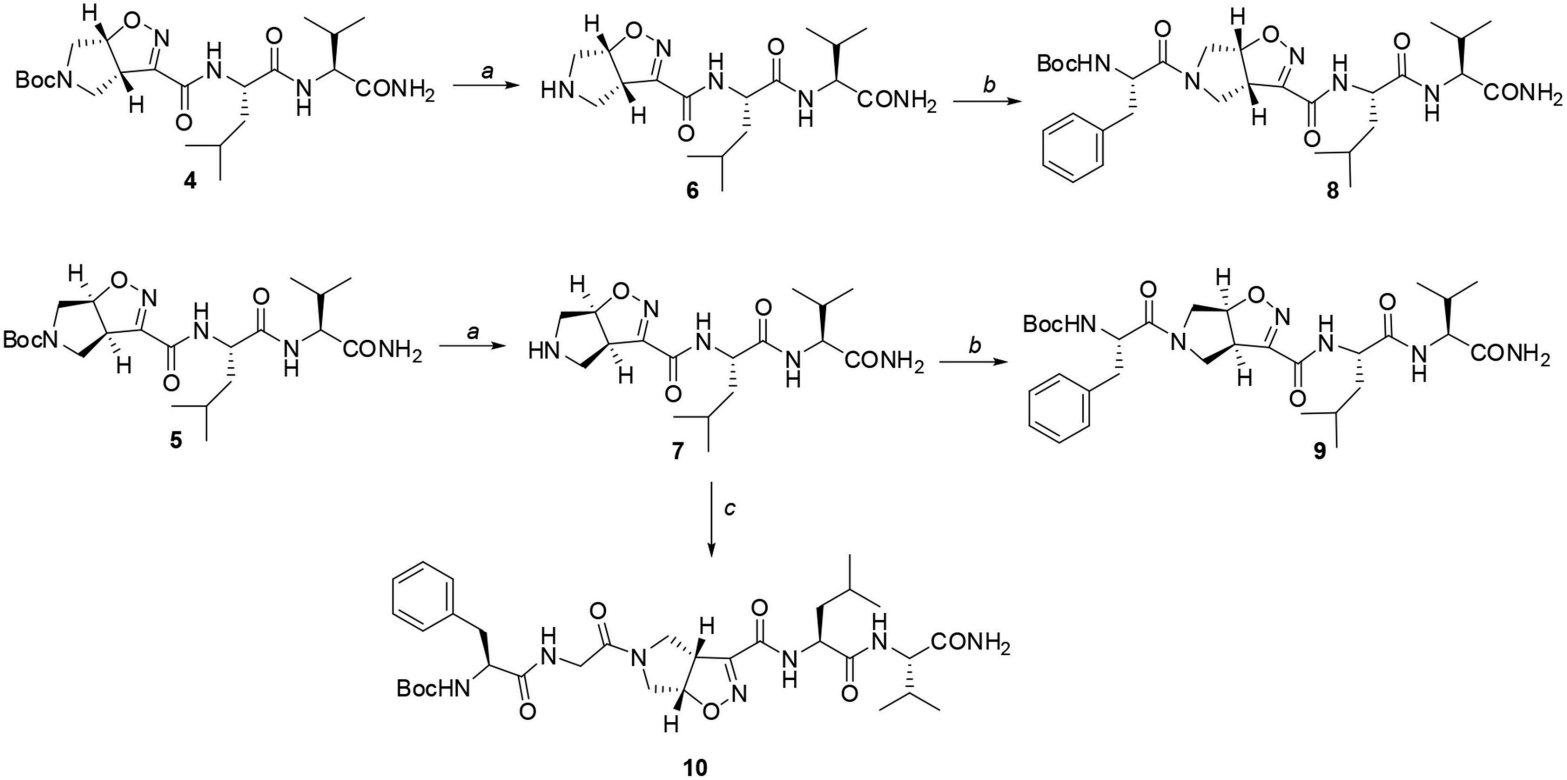
Reagents and conditions: a) TFA, CH_2_Cl_2_ from 0°C to r.t, 2 h; b) EDC/HOBt/DIEA, *N*-Boc-(L)-Phe, CH_2_Cl_2_ from 0°C to r.t, 24 h; c) EDC/HOBt/DIEA, *N*-Boc-(L)-Phe-Gly-OH, CH_2_Cl_2_ from 0°C to r.t, 24 h.

The ability of compound **1** to stabilize secondary structures was evaluated in model peptides containing (l)-Phe at *N*-terminus and (l)-Leu-(l)-Val sequence at *C*-terminus and (Schemes 2, 3). This last dipeptide was chosen as it normally adopts extended conformation in solution. Compound **1** was used in both the 3a*S*6a*R* and 3a*R*6a*S* stereochemistries [(–)-**1** and (+)-**1**, respectively], as a different effect on peptide conformations could be expected depending on the stereochemistry of the unnatural amino acids (Pellegrino et al., 2012).

Firstly, Leu-Val dipeptide **3** was prepared starting from (l)-Valinamide and *NH*-Boc-(l)-Leu, according to the general coupling procedure, followed by *N*-terminus deprotection in TFA (91% overall yield). Diastereoisomeric dipeptides **4** and **5** were then achieved in good yields (65% and 63%, respectively) by a coupling reaction of (–)-**1** or (+)-**1** with dipeptide **3** (Scheme 2).

Both **4** and **5** compounds were completely characterized by NMR spectroscopy (CDCl_3_, see SI for complete proton assignment). In both tripeptides, no significant Noesy effects between Leu-Val dipeptide and the scaffold were detected. Furthermore, in variable temperature experiments, the obtained Δδ/ΔT is higher than 5 ppb/K for all the amide protons, indicating the absence of H-bonds stabilizing a particular conformation. The *J*_NH−CHα_ value for NH-Val is of 8.8 Hz and 7.9 Hz, for **4** and **5**, respectively (NH-Leu appears as a broad signal for each compound). From these findings, we hypothesized that both **4** and **5** adopt an extended conformation and that the isoxazoline scaffold is not interacting with Leu-Val dipeptide.

The peptide chain was then elongated at *N*-terminus, through Boc-deprotection and coupling with (l)-Phe. Diastereoisomeric dipeptides **8** and **9** were achieved in good overall yields (73% and 76%, respectively) (Scheme 3). From NMR studies, it was found that both model peptides **8** and **9** are present as a mixture of conformers in 2:1 ratio in CDCl_3_ solution. The presence of these two conformers is probably due to the low-energy barrier *cis/trans* isomerization of the tertiary amide on the pyrrolidine as it is frequently observed on the acylated proline and in the case of tertiary cyclic amides (Laursen et al., 2013; Pellegrino et al., 2014). Regarding compound **8**, this rotation led to a splitting of the (l)-Phe proton signals only. Furthermore, no significative Noesy effects were detected, and, in variable temperature experiments, the obtained Δδ/ΔT coefficient is high for all the amide protons. Taking all these data together, we can assume that Phe and Leu-Val dipeptide are oriented in opposite directions and that the 3a*S*6a*R* stereochemistry of the scaffold is not effective in inducing specific secondary structures when used in combination with (l)-α-amino acids.

**Figure 2:**
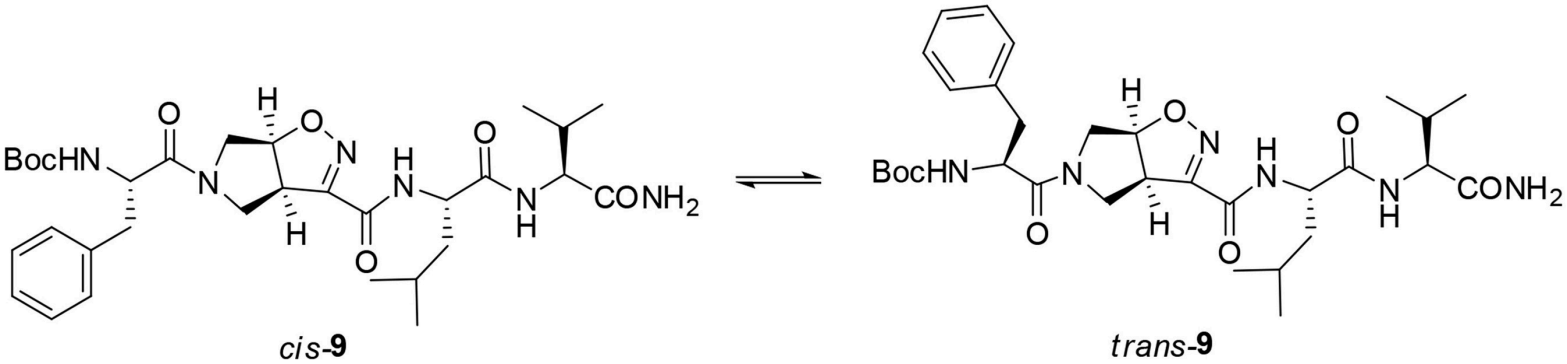
*Cis* and *trans* isomers of compound **9**.

A different scenario was observed for compound **9**. Its two rotamers are indeed characterized by different chemical shifts, suggesting that the isomerization of the tertiary amide leads to two different structures conformations influencing the entire molecule. Unfortunately, due to several overlapping signals, it was not possible to assessing significative Noesy proximities. Variable temperature experiments were thus done, in order to investigate if the NH were involved in hydrogen bonds and, as a consequence, if the two isomers of **9** were characterized by a stable conformation in solution. In the case of the major isomer, the obtained Δδ/ΔT is around 4 ppb/K for NH-Phe, indicating that this proton could be involved in a weak/medium hydrogen bond. All the other amide protons had higher Δδ/ΔT. On the other hand, in the minor conformer the obtained Δδ/ΔT is of around 2 ppb/K for NH-Val, indicating that this proton is involved in a strong hydrogen bond. Metadynamic studies were then performed on the cis/trans tertiary amide conformers of compound **9** (Figure 2).

**Figure 3:**
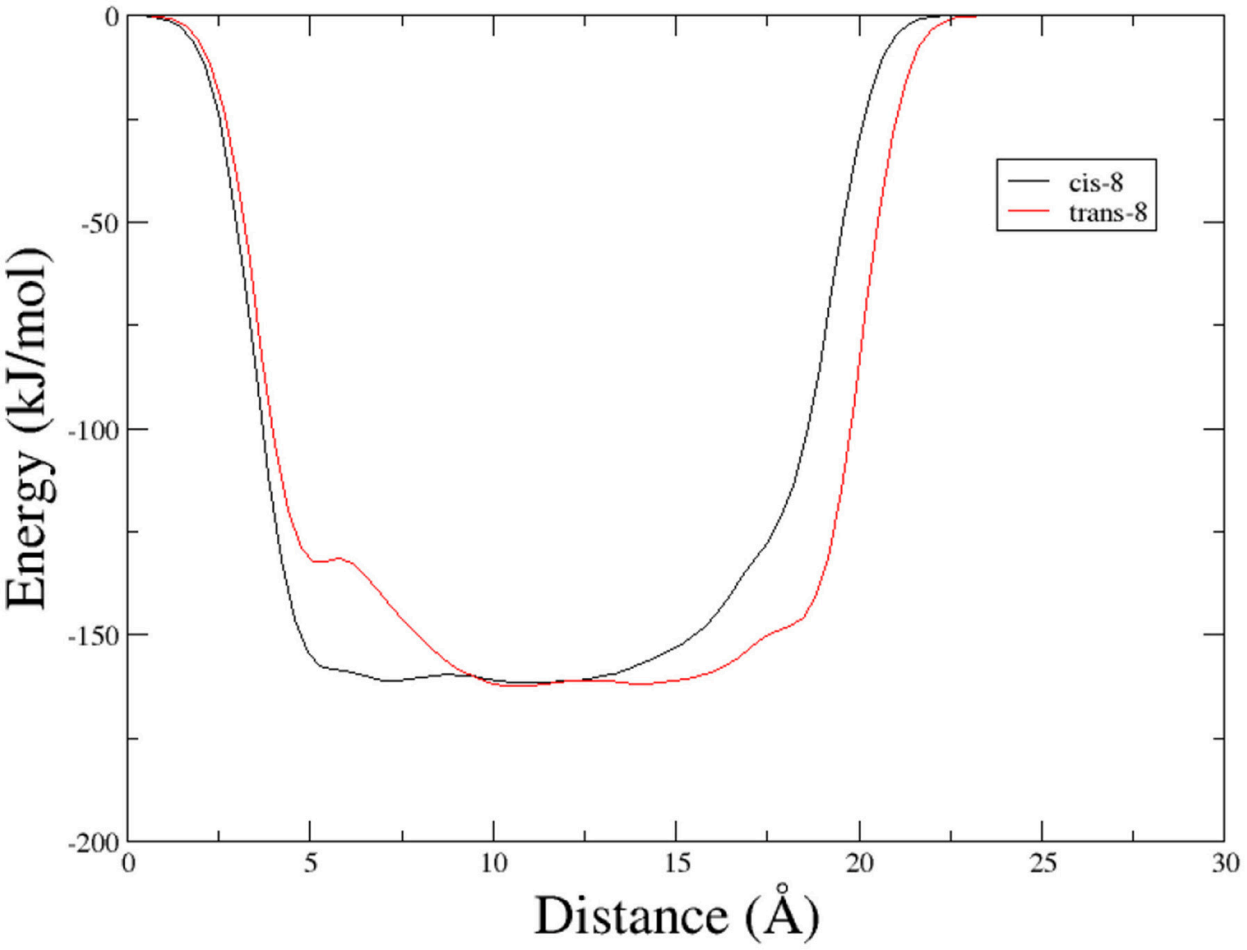
Potential energy as a function of distance between Boc quaternary carbon and C_β_Val obtained from Metadynamics for the two isomers of compound **9** in chloroform.

In particular, we performed two 50 ns long metadynamics simulations, using the distance between Boc quaternary carbon and C_β_Val as collective variable (CV). This geometric parameter was selected as a suitable CV because it discriminates well between closed and extended states of the peptide. In this way, it could be possible to evaluate if the two conformers had an intrinsic tendency toward turn conformation. The *cis* isomer showed a broad free energy minimum corresponding to CV values between 5 and 8 Å, while the *trans* isomer showed much higher free energy values in this region, exhibiting a shallow minimum around 10–15 Å (Figure 3).

**Figure 4:**
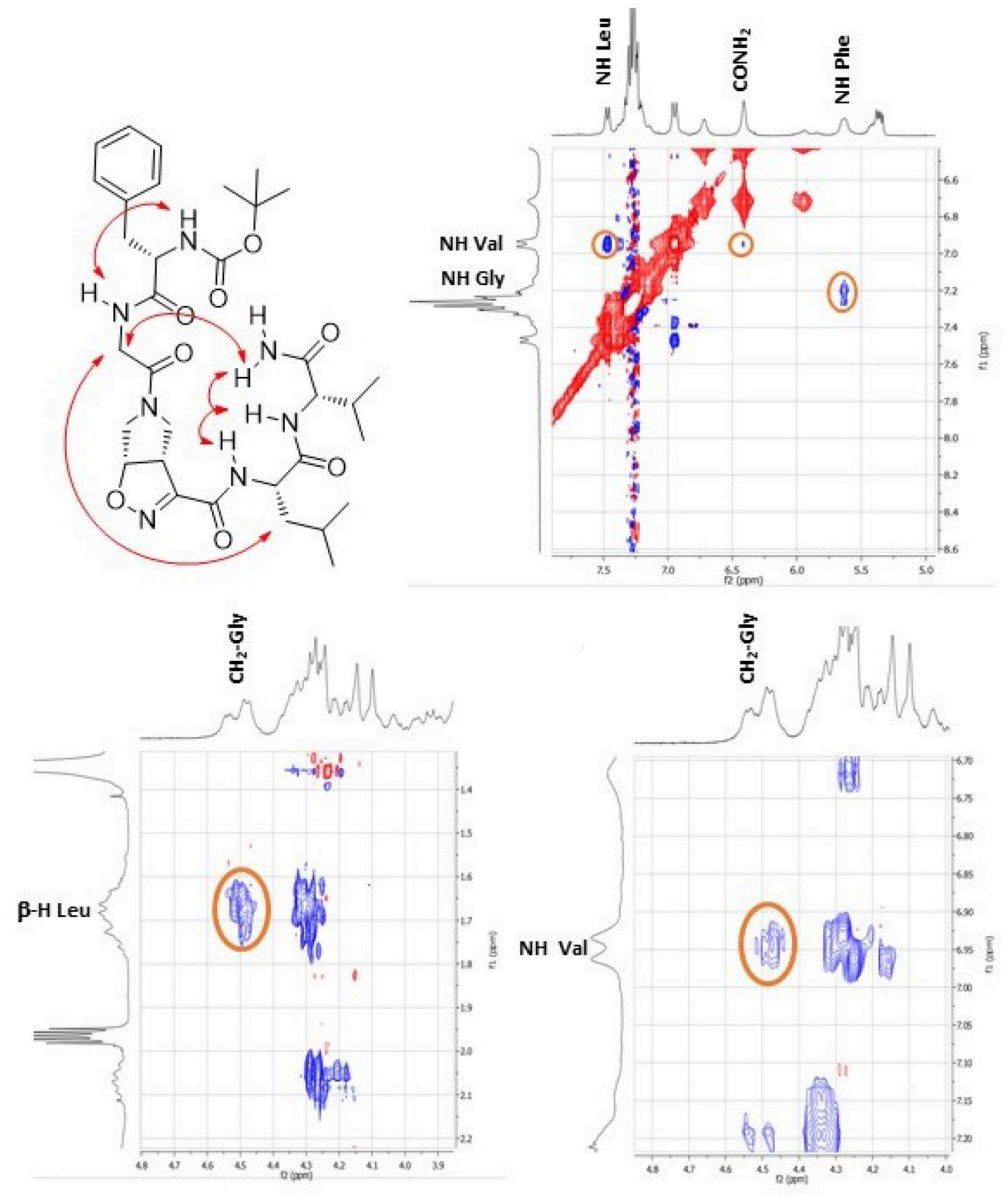
Noesy proximity (red) observed for compound **10** (CD_3_CN). On the top right: zoom of the amide region. On the bottom left: Zoom of the aliphatic region. On the bottom right: zoom of the NH-aliphatic region.

This different behavior could be indicative of a preference of *cis*-**9** toward a more closed conformation, although from these data it was not possible to make any further assumptions. For this reason, we envisaged that the introduction of a Gly residue between Phe and isoxazoline scaffold could increase the conformational flexibility of the *N*-terminus peptide arm favoring its interaction with the *C*-teminus part of the molecule. Compound **7** was thus elongated at *N*-terminus, through coupling with *N*-Boc-(l)-Phe-Gly-OH dipeptide using general coupling reaction conditions to obtain peptide **10** (65%, Scheme 3). The NMR study on **10** (CD_3_CN, see SI for complete assignment), showed that it is present in solution as a single stable conformer (only trace amounts of a second isomer were detected). A complete set of NH_i_-NH_i+1_ Noesy proximities, whose calculated distances ranged from 2.68 to 2.96 Å, were observed (Figure 4 on the top right). Furthermore, long range Noesy effects involving HαGly and NHVal (3.03 Å) and HβLeu (2.73 Å) were found (Figure 4 on the bottom).

**Figure 5:**
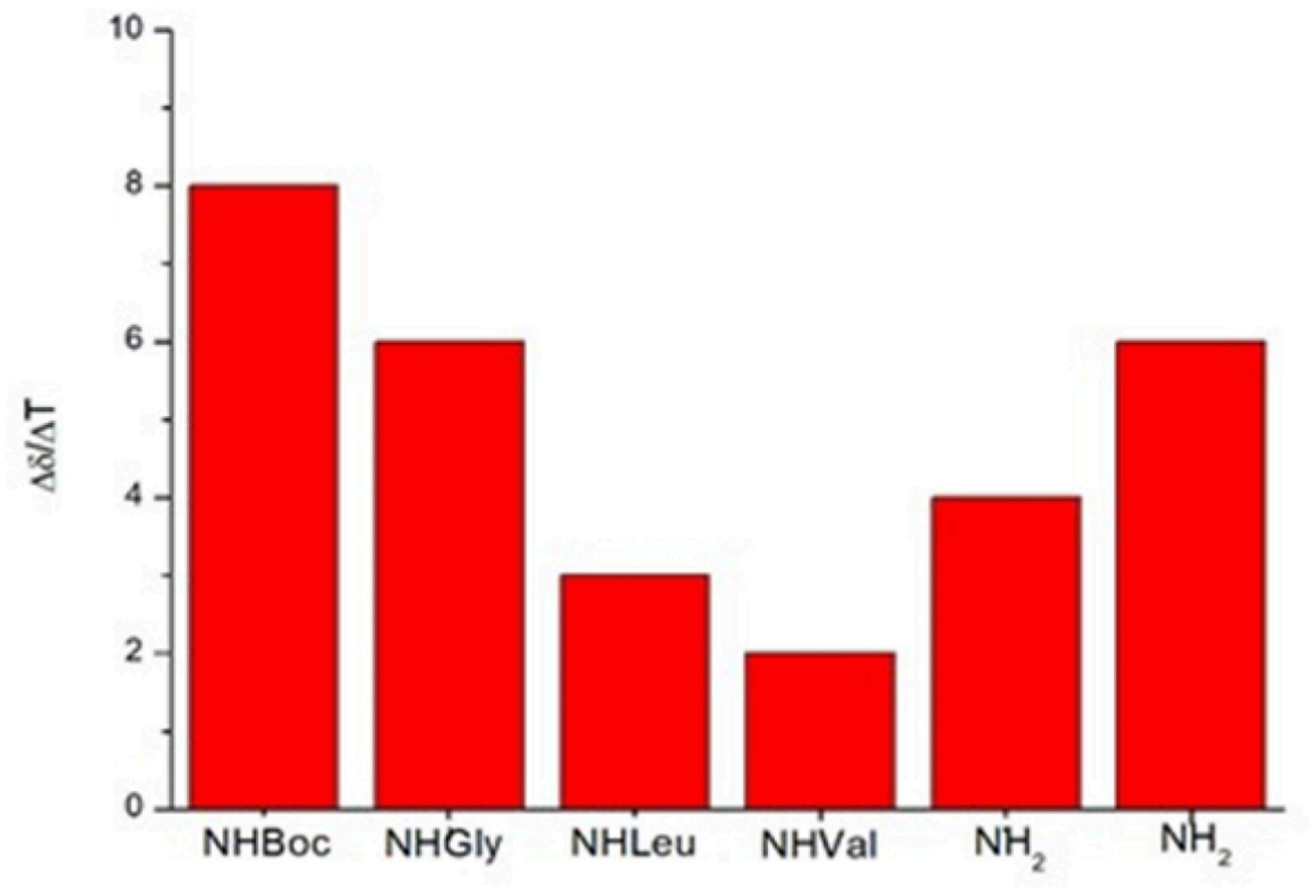
Variable temperature coefficients of the amide protons (CD_3_CN).

In variable temperature experiments (Figure 5), the obtained Δδ/ΔT is of around 2 ppb/K for NH-Val, while NH-Leu and one of the C-terminus amide protons possess Δδ/ΔT of around 3 and 4 ppb/K, respectively. The presence of hydrogen bonds was also confirmed by FT-IR analysis. N-H stretching bands A and B (3,500–3,000 cm^−1^region, Figure S20) are indeed downshifted as frequently observed in intramolecular hydrogen bonded conformations (Tonan and Ikawa, 1996; Barth, 2007). Furthermore, the deconvolution of the amide I band (1,700–1,600 cm^−1^) showed the presence of a band at 1,655 cm^−1^ (Figure S20), typical of α structures (Kong and Yu, 2007).

**Figure 6:**
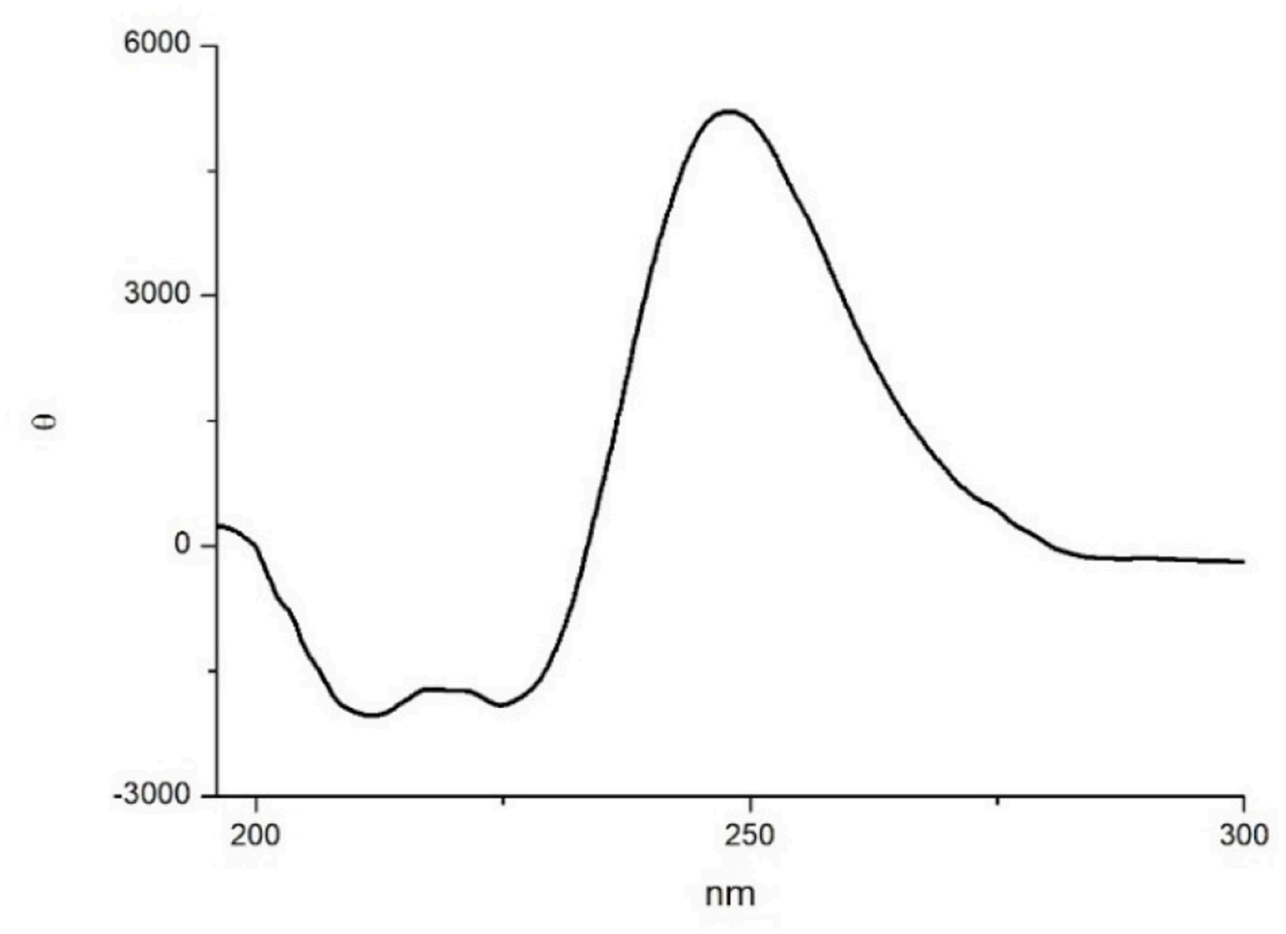
CD spectrum of compound **10** in CH_3_CN (0.1 mM CH_3_CN solution).

In order to gain more information on the conformation of compound **10**, far-UV circular dichroism (CD) analysis in CH_3_CN (0.1 mM solution) was then performed. In Figure 6 the obtained CD spectrum is reported.

It showed a maximum at around 250 nm due to the strong absorption of the isoxazoline ring, as observed by Memeo et al. (2018) on similar compounds (see also the UV spectrum in the SI). In the amide bond region, two negative minima at 225 and 208 nm, with the same intensity, and a slightly positive band at 200 nm were also observed. The presence of the exciton splitting of the π → π^*^ transition band suggested that a α-turn conformation was present (Wang et al., 2018), although in short peptides it is difficult to correctly evaluate the intensity of Cotton effects with respect to secondary structure motifs (Chin et al., 2002; Wang et al., 2018).

**Figure 7:**
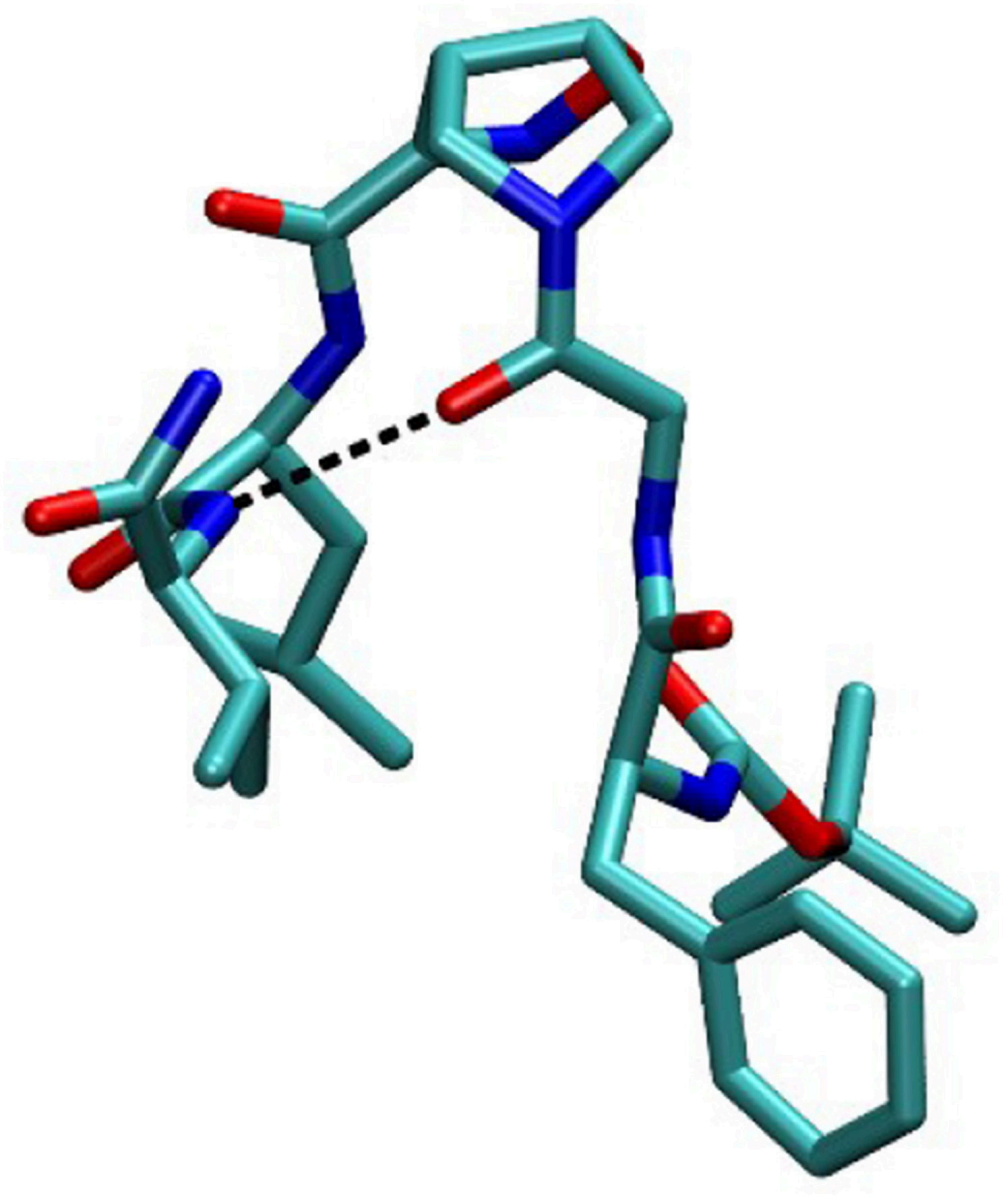
Compound **10** conformation during the restained MD.

In order to completely elucidate the conformation of peptide **10**, a restrained molecular dynamic was finally performed. The restraints were based on the obtained NOESY values. When the hydrogen was not uniquely defined, like in H_α_Gly and H_β_Leu, we used the closest carbon, e.g., C_α_Gly and C_β_Leu to implement distance restrains (Figure S3). The analysis showed that C_α_Gly-C_β_Leu (A) and C_α_Gly-H_NH_Val (B) are mutually exclusive in the *trans* isomer but not in the *cis one* as shown in Figure S1. Furthermore, H-bond analysis resulted in the observation that a H-bond is between CO-Gly and NH-Val. In Figure 7 the most representative structure is reported.

Taking together both experimental and molecular modeling data, we can assume that, in solution, compound **10** effectively adopts a α-turn conformation. This motif is normally formed by 5 α-amino acids and is characterized by a H-bond between *i* and *i*+*4* residues. In our case, the isoxazoline scaffold replaces two of the three core amino acids (Figure 1) and the H-bond is formed by the CO-Gly and NH-Val, leading to the formation of a 12 member pseudo-cycle. Furthermore, the overall structure of the peptidomimetic is reinforced by medium-weak H-bonds involving NH-Leu and one C-terminus-NH_2_, as evicted from NMR data. The presence of a α-turn conformation is also confirmed by the computational analysis of the dihedral angles of the residue i+3 and of the distances between residues i and i+4 (Table S9), as their values rely within the α-turn parameters (Pavone et al., 1996).

In conclusion, as α-turns are often found on biologically active sites and few molecules have been reported to mimic or stabilize them, the ability of compound (+)-**1** to stabilize α-turn conformation on isolated peptide is particularly important in view of future biological applications.

## Data Availability

All datasets generated for this study are included in the manuscript and/or the supplementary files.

## Author Contributions

FO and RB equally contributed to this work. The project was conceived by SaP, LT, and AP. SaP designed and coordinated the research. LT and RB chemically synthesized and analyzed the materials, RB performed NMR, FT-IR and CD experiments, FO and StP performed molecular modeling. SaP, StP, LT, and AP analyzed and compiled the data and co-wrote the manuscript. The final manuscript was read and approved by all the authors.

### Conflict of Interest Statement

The authors declare that the research was conducted in the absence of any commercial or financial relationships that could be construed as a potential conflict of interest.

## Abbreviations

ACN: Acetonitrile
Boc: tert-butyloxycarbonyl
DIEA, N: N-Diisopropylethylamine
DMF: Dimethylformamide
EDC: 1-Ethyl-3-(3-dimethylaminopropyl)carbodiimide
Gly: Glycine
HOBt: Hydroxybenzotriazole
Leu: Leucine
Phe: Phenylalanine
TFA: Trifluoroacetic acid
Val: Valine.

## References

[B1] Abraham M. Van Der Spoel D. Lindahl E. Hess B. (2014). GROMACS User Manual Version 5.0.4. Available online at: https://ftp.gromacs.org/pub/manual/manual-5.0.4.pdf

[B2] Barth A. (2007). Infrared spectroscopy of proteins. BBA Bioenerg. 1767, 1073–1101. 10.1016/j.bbabio.2007.06.004 17692815

[B3] Berendsen H. J. C. Postma J. P. M. van Gusteren W. F. DiNola A. (1984). Molecular dynamics with coupling to an external bath. J. Chem. Phys. 81, 3684–3690. 10.1063/1.448118

[B4] Bonetti A. Pellegrino S. Das P. Yuran S. Bucci R. Ferri N. (2015). Dipeptide Nanotubes Containing Unnatural Fluorine-Substituted β2,3-Diarylamino Acid and l-Alanine as candidates for biomedical applications. Org. Lett. 17, 4468–4471. 10.1021/acs.orglett.5b02132 26335611

[B5] Bouillère F. Thétiot-Laurent S. Kouklovsky C. Alezra V. (2011). Foldamers containing γ-amino acid residues or their analogues: structural features and applications. Amino Acids 41, 687–707. 10.1007/s00726-011-0893-3 21455734

[B6] Bruno S. Pinto A. Paredi G. Tamborini L. De Micheli C. La Pietra V.. (2014). Discovery of covalent inhibitors of glyceraldehyde-3-phosphate dehydrogenase, a target for the treatment of malaria. J. Med. Chem. 57, 7465–7471. 10.1021/jm500747h 25137375

[B7] Bucci R. Das P. Iannuzzi F. Feligioni M. Gandolfi R. Gelmi M. L.. (2017). Self-assembly of an amphipathic α*αβ*-tripeptide into cationic spherical particles for intracellular delivery. Org. Biomol, Chem. 15, 6773–6779. 10.1039/C7OB01693J 28767120

[B8] Bucci R. Giofré S. Clerici F. Contini A. Pinto A. Erba E. (2018). Tetrahydro-4H-(pyrrolo[3,4-*d*]isoxazol-3-yl)methanamine: a bicyclic diamino scaffold stabilizing parallel turn conformations. *J*. Org. Chem. 83, 11493–11501. 10.1021/acs.joc.8b01299 30192540

[B9] Bussi G. Donadio D. Parrinello M. (2007). Canonical sampling through velocity rescaling. J. Phys Chem. 126:014101. 10.1063/1.2408420 17212484

[B10] Castellano S. Kuck D. Viviano M. Yoo J. López-Vallejo F. Conti P.. (2011). Synthesis and biochemical evaluation of δ 2-isoxazoline derivatives as DNA methyltransferase 1 inhibitors. J. Med. Chem. 54, 7663–7677. 10.1021/jm2010404 21958292

[B11] Chatterjee S. Vasudev P. G. Raghothama S. Ramakrishnan C. Shamala N. Balaram P. (2009). Expanding the peptide β-turn in αγ hybrid sequences: 12 atom hydrogen bonded helical and hairpin turns. J. Am. Chem. Soc. 131, 5956–5965. 10.1021/ja900618h 19341285

[B12] Chin D.-H. Woody R. W. Rohl C. A. Baldwin R. L. (2002). Circular dichroism spectra of short, fixed-nucleus alanine helices. Proc. Natl. Acad. Sci. U.S.A. 99, 15416–15421. 10.1073/pnas.232591399 12427967 PMC137731

[B13] Clerici F. Erba E. Gelmi M. L. Pellegrino S. (2016). Non-standard amino acids and peptides: from self-assembly to nanomaterials Tetrahedron Lett. 57, 5540–5550. 10.1016/j.tetlet.2016.11.022

[B14] Conti P. De Amici M. Pinto A. Tamborini L. Grazioso G. Frolund B. (2006). Synthesis of 3-hydroxy- and 3-carboxy-Δ2-isoxazoline amino acids and evaluation of their interaction with GABA receptors and transporters. Eur. J. Org. Chem. 24, 5533–5542. 10.1002/ejoc.200600628

[B15] Conti P. Pinto A. Wong P. E. Major L. L. Tamborini L. Iannuzzi M. C.. (2011). Synthesis and *in vitro*/*in vivo* evaluation of the antitrypanosomal activity of 3-bromoacivicin, a potent CTP synthetase inhibitor. ChemMedChem 6, 329–333. 10.1002/cmdc.201000417 21275056 PMC3504386

[B16] Fanelli R. Berthomieu D. Didierjean C. Doudouh A. Lebrun A. Martinez J.. (2017). Hydrophobic α,α-disubstituted disilylated TESDpg induces incipient 310-Helix in short tripeptide sequence. Org. Lett. 19, 2937–2940. 10.1021/acs.orglett.7b01172 28514165

[B17] Fuller A. Chen B. Minter A. R. Mapp A. K. (2005). Succinct synthesis of β-amino acids via chiral isoxazolines. J. Am. Chem. Soc. 127, 5376–5383. 10.1021/ja0431713 15826175

[B18] Gandini E. Dapiaggi F. Oliva F. Pieraccini S. Sironi M. (2018) Well-Tempered MetaDynamics based method to evaluate universal peptidomimetics. Chem. Phys. Lett. 706, 729–735. 10.1016/j.cplett.2018.07.029

[B19] Guo L. Zhang W. Guzei I. A. Spencer L. C. Gellman S. H. (2012). New preorganized γ-amino acids as foldamer building blocks. Org. Lett. 14, 2582–2585. 10.1021/ol3008815 22568480 PMC3445736

[B20] Hoang H. N. Driver R. W. Beyer R. L. Malde A. K. Le G. T. Abbenante G (2011). Protein α-turns recreated in structurally stable small molecules. Angew Chem. Int. Ed. 50, 11107–11111. 10.1002/anie.201105119 21956852

[B21] Hornak V. Abel R. Okur A. Strockbine B. Roitberg A. Simmerling C. (2006). Comparison of multiple Amber force fields and development of improved protein backbone parameters. Proteins 65, 712–725. 10.1002/prot.21123 16981200 PMC4805110

[B22] Itoh T. Watanabe M. Fukuyama T. (2002). Synthetic approach to tetrodotoxin. Synlett 8, 1323–1325. 10.1055/s-2002-32984

[B23] Kaur K. Kumar V. Sharma A. K. Gupta G. K. (2014). Isoxazoline containing natural products as anticancer agents: a review. Eur. J. Med. Chem. 77, 121–133. 10.1016/j.ejmech.2014.02.063 24631731

[B24] Kelso M. J. Beyer R. L. Hoang H. N. Lakdawala A. S. Snyder J. P. Oliver W. V.. (2004). Alpha-turn mimetics: short peptide alpha-helices composed of cyclic metallopentapeptide modules. J Am. Chem. Soc. 126, 4828–4842. 10.1021/ja037980i 15080687

[B25] Kobayashi H. Misawa T. Matsuno K. Demizu Y. (2017). Preorganized cyclic α,α-disubstituted α-amino acids bearing functionalized side chains that act as peptide-helix inducers. *J*. Org. Chem. 82, 10722–10726. 10.1021/acs.joc.7b01946 28915041

[B26] Konda M. Jadhav R. G. Maiti S. Mobin S. M. Kauffmannb B. Das A. K. (2018). Understanding the conformational analysis of gababutin based hybrid peptides. Org. Biomol. Chem. 16, 1728–1735. 10.1039/C8OB00035B 29457824

[B27] Kong J. Yu S. (2007). Fourier transform infrared spectroscopic analysis of protein secondary structures. Acta Biochim. Biophys. Sinica. 39, 549–559. 10.1111/j.1745-7270.2007.00320.x 17687489

[B28] Kozikowski A. P. Park P. U. (1990). Synthesis of streptazolin: use of the aza-Ferrier reaction in conjunction with the INOC process to deliver a unique but sensitive natural product J. Org. Chem. 55, 4668–4682. 10.1021/jo00302a036

[B29] Krishna Y. Sharma S. Ampapathi R. S. Koley D. (2014). Furan-based locked Z-vinylogous γ-amino acid stabilizing protein α-turn in water-soluble cyclic α3γ tetrapeptides. Org. Lett. 16, 2084–2087. 10.1021/ol5002126 24697707

[B30] Laursen J. S. Engel-Andreasen J. Fristrup P. Harris P. Olsen C. A. (2013). Cis–trans amide bond rotamers in β-peptoids and peptoids: evaluation of stereoelectronic effects in backbone and side chains. *J*. Am. Chem. Soc. 135, 2835–2844. 10.1021/ja312532x 23343406

[B31] López-Andarias A. López-Andarias J. Atienza C. Chichón F. J. Carrascosa J. L. Martín N. (2018). Tuning optoelectronic and chiroptic properties of peptide-based materials by controlling the pathway complexity. Chem. Eur. J. 24, 7755–7760. 10.1002/chem.201801238 29537693

[B32] Machetti F. Ferrali A. Menchi G. Occhiato E. G. Guarna A. (2000). Oligomers of enantiopure bicyclic γ/δ-amino acids (BTAa). 1. Synthesis and conformational analysis of 3-aza-6,8-dioxabicyclo[3.2.1]octane-7-carboxylic acid oligomers (PolyBTG). Org. Lett. 2, 3987–3990. 10.1021/ol006548s 11112624

[B33] Memeo M. G. Bruschi M. Bergonzi L. Desimoni G. Faita G. Quadrelli P. (2018). Cyclopenta[d]isoxazoline β-turn mimics: synthetic approach, turn driving force, scope, and limitations. ACS Omega 3, 13551–13558. 10.1021/acsomega.8b01670 PMC6645019 31458062

[B34] Mikhalevich V. Craciun I. Kyropoulou M. Palivan C. G. Meier W. (2017). Amphiphilic peptide self-assembly: expansion to hybrid materials. Biomacromolecules 18, 3471–3480. 10.1021/acs.biomac.7b00764 28776980

[B35] Pavone V. Gaeta G. Lombardi A. Nastri F. Maglio O. Isernia C.. (1996). Discovering protein secondary structures: classification and description of isolated α-turns. Biopolymers 38, 705–721. 10.1002/(SICI)1097-0282(199606)38:6<705::AID-BIP3>3.0.CO;2-V 8652792

[B36] Pellegrino S. Bonetti A. Clerici F. Contini A. Moretto A. Soave R.. (2015). 1H-azepine-2-oxo-5-amino-5-carboxylic acid: a 310 helix inducer and an effective tool for functionalized gold-nanoparticles. J. Org. Chem, 80, 5507–5516. 10.1021/acs.joc.5b00396 25938852

[B37] Pellegrino S. Contini A. Clerici F. Gori A. Nava D. Gelmi M. L. (2012). 1H-Azepine-4-amino-4-carboxylic acid: a new α,α disubstituted ornithine analogue capable of inducing helix conformations in short Ala-Aib pentapeptides. Chem. Eur. J. 18, 8705–8715. 10.1002/chem.201104023 22689465

[B38] Pellegrino S. Contini A. Gelmi M. L. Lo Presti L. Soave R. Erba E. (2014). Asymmetric modular synthesis of a semirigid dipeptide mimetic by cascade cycloaddition/ring rearrangement and borohydride reduction. J. Org. Chem, 79, 3094–3102. 10.1021/jo500237j 24635115

[B39] Pellegrino S. Facchetti G. Contini A. Gelmi M. L. Erba E. Gandolfi R. (2016). Ctr-1 Mets7 motif inspiring new peptide ligands for Cu(I)-catalyzed asymmetric Henry reaction under green conditions. RSC Adv. 6, 71529–71533. 10.1039/C6RA16255J

[B40] Pellegrino S. Tonali N. Erba E. Kaffy J. Taverna M. Contini A. (2017). ß-Hairpin mimics containing a piperidine-pyrrolidine scaffold modulate the ß-amyloid aggregation process preserving the monomer species. Chem. Sci. 8, 1295–1302. 10.1039/C6SC03176E 28451272 PMC5359901

[B41] Pinto A. Conti P. Grazioso G. Tamborini L. Madsen U. Nielsen B.. (2011). Synthesis of new isoxazoline-based acidic amino acids and investigation of their affinity and selectivity profile at ionotropic glutamate receptors. Eur. J. Med.Chem. 46, 787–793. 10.1016/j.ejmech.2010.12.020 21220180

[B42] Pinto A. Tamborini L. Cullia G. Conti P. De Micheli C. (2016a). Inspired by nature: the 3-halo-4,5-dihydroisoxazole moiety as a novel molecular warhead for the design of covalent inhibitors. ChemMedChem 1, 10–14. 10.1002/cmdc.201500496 26607551

[B43] Pinto A. Tamborini L. Pennacchietti E. Coluccia A. Silvestri R. Cullia G. (2016b). Bicyclic γ-amino acids as inhibitors of γ-aminobutyrate aminotransferase. J. Enzyme Inhib. Med. Chem. 2, 295–301. 10.3109/14756366.2015.1021251 25807299

[B44] Raymond D. M. Nilsson B. L. (2018). Multicomponent peptide assemblies. Chem. Soc. Rev. 47, 3659–3720. 10.1039/C8CS00115D 29697126 PMC6538069

[B45] Smith E. L. Spackman D. H. (1955). Leucine aminopeptidase. V. activation, specificity, and mechanism of action. J. Biol. Chem. 122, 271–299. 13233230

[B46] Sohora M. Vazdar M. Sović I. Mlinarić-Majerski K. Basarić N. (2018). Photocyclization of tetra- and pentapeptides containing adamantylphthalimide and phenylalanines: reaction efficiency and diastereoselectivity. J. Org. Chem. 83, 14905–14922. 10.1021/acs.joc.8b01785 30460849

[B47] Solomon L. A. Kronenberg J. B. Fry H. C. (2017). Control of heme coordination and catalytic activity by conformational changes in peptide–amphiphile assemblies. *J*. Am. Chem. Soc. 139, 8497–8507. 10.1021/jacs.7b01588 28505436

[B48] Sperry J. B. Wright D. L. (2005). Furans, thiophenes and related heterocycles in drug discovery. Curr. Opin. Drug Discov. Dev. 8, 723–740. 10.1002/chin.200615242 16312148

[B49] Steinke D. Kula M. R. (1990). Selektive desamidierung von peptidamiden. Angew Chemie 102, 1204–1206. 10.1002/ange.19901021035

[B50] Tamborini L. Mastronardi F. Dall'Oglio F. De Micheli C. Nielsen B. Lo Presti L. (2015). Synthesis of unusual isoxazoline containing β and γ-dipeptides as potential glutamate receptor ligands MedChemComm 6, 1260–1266. 10.1039/C5MD00159E

[B51] Tonan K. Ikawa S. (1996). Intramolecular hydrogen bonding and conformation of small peptides: variable-temperature FTIR study on N-Acetyl-l-Pro-l-Leu-Gly-NH_2_ and related compounds. J. Am. Chem Soc. 118, 6960–6965. 10.1021/ja953380a

[B52] Tribello G. A. Bonomi M. Branduardi D. Camilloni C. Bussi G. (2014). PLUMED 2: new feathers for an old bird. Comp. Phys. Comm. 185, 604–613. 10.1016/j.cpc.2013.09.018

[B53] Tsantali G. G. Dimtsas J. Tsoleridis C. A. Takakis I. M. (2007). Preparation of sixteen 3-hydroxy-4- and 7-hydroxy-1-hydrindanones and 3-hydroxy-4- and 8-hydroxy-1-hydroazulenones. Eur. J. Org. Chem. 2007, 258–265. 10.1002/ejoc.200600639

[B54] Van Der Spoel D. Lindahl E. Hess B. Groenhof G. Mark A. E Berendsen H. J. (2005). GROMACS: fast, flexible, and free. J. Comput. Chem. 26, 1701–1718. 10.1002/jcc.20291 16211538

[B55] Vasudev P. G. Chatterjee S. Shamala N. Balaram P. (2011). Structural chemistry of peptides containing backbone expanded amino acid residues: conformational features of β, γ, and hybrid peptides. Chem. Rev. 111, 657–687. 10.1021/cr100100x 20843067

[B56] Wang L. Coric P. Zhu K. Liu W.-Q. Vidal M. Bouaziz S.. (2018). Synthesis and characterization of water-soluble macrocyclic peptides stabilizing protein α-turn. Org. Biomol. Chem. 16, 459–471. 10.1039/C7OB02852K 29265149

[B57] Wintjens R. T. Rooman M. J. Wodak S. J. (1996). Automatic classification and analysis of alpha alpha-turn motifs in proteins. J. Mol. Biol. 255, 235–253. 10.1006/jmbi.1996.0020 8568871

[B58] Zhang X. X. Eden H. S. Chen X. (2012). Peptides in cancer nanomedicine: drug carriers, targeting ligands and protease substrates. J. Control Release 159, 2–13. 10.1016/j.jconrel.2011.10.023 22056916 PMC3288222

