# Supplementary material for: Bicyclic Pyrrolidine-Isoxazoline γ Amino Acid: A Constrained Scaffold for Stabilizing α-Turn Conformation in Isolated Peptides
Source: Front Chem. 2019 Mar 18;7:133. doi: 10.3389/fchem.2019.00133 (PMC6431668; doi:10.3389/fchem.2019.00133)
Supplement: Supplementary file 1 [file Table_1.DOCX]

**Bicyclic pyrrolidine-isoxazoline γ amino acid: a constrained scaffold for stabilizing α-turn conformation in isolated peptides.**

Francesco Oliva, Raffaella Bucci, Lucia Tamborini, Stefano Pieraccini, Andrea Pinto, Sara Pellegrino

**Supporting Information**

**^1^H-NMR characterization of compounds 4, 5, 8-10 2**

**^1^H-NMR and ^13^C-NMR spectra of compounds 1, 4-10 10**

**UV spectrum of compound 10 28**

**FT-IR NH-stretching and amide I regions 29**

**Molecular modelling 30**

**^1^H-NMR characterization of compounds 4, 5, 8-10**

*Compound* ***4***

**Table S1**. NMR Characterization of compound **4** (CDCl_3_, 28 mM, 293 K, 300 MHz)

| ***Residue*** | ***NH*** | ***Hα*** | ***Hβ*** | ***Other*** | ***Noesy*** |
| --- | --- | --- | --- | --- | --- |
| *t*BuCO | - | - | - | 1.43 (s) |  |
| scaffold | - | - | H3a: 5.34-5.29 (m)  H6a: 4.16-4.03 (m)  H4: 4.05-3.92 (m, overl.)  3.57-3.36 (m, overl.)  H6: 3.95-3.83,  3.57-3.36 (m, overl.) | |  |
| Leu | 7.11 (bs) | 4.56-4.48 (m) | 1.75-1.71 (m, overl.) | 1.75-1.71 (m,overl.)  0.99-0.92 (m, overl.) |  |
| Val | 6.88 (d, *J=*8.8 Hz) | 4.31 (dd, *J*=8.2,  *J*=6.9) | 2.18-2.06 (m) | 0.99-0.92 (m, overl.) |  |
| CONH_2_ | 6.19 (bs)  5.72 (bs) | - | - | - |  |

*Compound* ***5***

6a

6

4

| ***Residue*** | ***NH*** | ***Hα*** | ***Hβ*** | ***Other*** | ***Noesy*** |
| --- | --- | --- | --- | --- | --- |
| *t*BuCO | - | - | - | 1.49 (s) |  |
| scaffold | - | - | H3a: 5.34-5.26 (m)  H6a: 4.18-4.05 (m),  H4: 3.99-3.84 (m)  3.54-3.48 (m, overl.)  H6: 3.54-3.48 (m, overl.) | |  |
| Leu | 7.05 (bs) | 4.57-4.46 (m) | 1.69-1.64 (m, overl.) | 1.69-1.64 (m, overl.)  1.08-0.88 (m, overl.) | NH-HαVal |
| Val | 6.73 (d, *J=* 7.9 Hz) | 4.33-4.28 (m) | 2.24-2.12 (m) | 1.08-0.88 (m, overl.) | Hα-NHLeu |
| CONH_2_ | 6.19 (bs)  5.72 (bs) | - | - | - |  |

**Table S2**. NMR Characterization of compound **5** (CDCl_3_, 28 mM, 293 K, 300 MHz)

*Compound* ***8*** *2 conformers (2:1)*

**Tables S3**. NMR Characterization of compound **8** major isomer (CDCl_3_, 22 mM, 293 K, 300 MHz)

| ***Residue*** | ***NH*** | ***α-H*** | ***β-H*** | ***Other*** |
| --- | --- | --- | --- | --- |
| tBuCO | - | - | - | 1.45 (s) |
| Phe | 5.43 (d, *J=*8.4 Hz) | 4.65-4.59 (m, overl.) | 3.09-3.05, 2.95-2.89 (m) | 7.30-7.23 (m, aromatic) |
| Scaffold |  |  | H3a: 5.07-4.97 (bs)  H4: 3.82-3.76 (m, overl.)  2.51 (dd,  *J*=12.2,  *J*=4.8)  H6: 4.20-4.15 (m, overl.)  3.40-3.33 (m)  H6a: 3.98-3.92 (m) | |
| Leu | 7.04-6.97 (m) | 4.54-4.63 (m, overl.) | 1.69-1.56 (m, overl.) | 1.69-1.56 (m, overl.)  0.99-0.92 (m, overl.) |
| Val | 6.87-6.77 (m) | 4.32-4.27 (m) | 2.20-2.15 (m) | 0.99-0.92 (m, overl.) |
| CONH_2_ | 6.05 (bs)  5.71 (bs) |  |  |  |

**Tables S4**. NMR Characterization of compound **8** minor isomer (CDCl_3_, 22 mM, 293 K, 300 MHz)

| ***Residue*** | ***NH*** | ***α-H*** | ***β-H*** | ***Other*** |
| --- | --- | --- | --- | --- |
| tBuCO | - | - | - | 1.40 (s) |
| Phe | 5.27-5.25 (m, overl.) | 4.51-4.46 (m, overl.) | 2.95-2.89 (m), 2.86-2.84 (m) | 7.28-7.23 (m, aromatic) |
| Scaffold |  |  | H3a: 5.35-5.20 (m, overl.)  H4: 4.20-4.15 (m, overl.)  3.64-3.58 (m)  H6: 3.82-3.76 (m, overl.)  3.69-3.67 (m, overl.)  H6a: 4.20-4.15 (m, overl.) | |
| Leu | 7.04-6.97 (m) | 4.54-4.63 (m, overl.) | 1.69-1.56 (m, overl.) | 1.69-1.56 (m, overl.)  0.99-0.92 (m, overl.) |
| Val | 6.87-6.77 (m) | 4.32-4.27 (m) | 2.20-2.15 (m) | 0.99-0.92 (m, overl.) |
| CONH_2_ | 6.05 (bs)  5.71 (bs) |  |  |  |

*Compound* ***9*** *2 conformers (2:1)*

**Tables S5**. NMR Characterization of compound **9** major isomer (CDCl_3_, 22 mM, 293 K, 300 MHz)

| ***Residue*** | ***NH*** | ***α-H*** | ***β-H*** | ***Other*** | ***Noesy*** | ***Δδ/ΔT*** |
| --- | --- | --- | --- | --- | --- | --- |
| tBuCOO |  | - | - | 1.44 (s) | - | - |
| Phe | 5.46 (d, *J*=8.4) | 4.82-4.75 (m) | 3.07-2.93 (m) | 7.28-7.23 (m, aromatic) |  | 3 |
| Scaffold |  |  | H3a: 5.22-5.17 (m, overl.)  H4: 3.93-3.86 (d, *J*=11.9)  2.54 (dd,  *J*=11.9,  *J*=8.5)  H6: 4.36-4.31 (m, overl.)  3.21 (dd,  *J*=14.3,  *J*=5.0)  H6a: 4.12-4.09 (m, overl.) | |  | - |
| Leu | 7.05 -6.93 (m, overl.) | 4.63-4.48 (m) | 1.86-1.57 (m overl.) | 1.86-1.57 (m, overl.)  0.91 (m, overl.) |  | Overl. |
| Val | 7.19 (m, overl.) | 4.36-4.31 (m, overl.) | 2.17-2.04 (m, overl.) | 0.91 (m, overl.) |  | >5 |
| NH_2_ | 6.08 bs  5.99 bs |  |  |  |  | >5 |

**Tables S6**. NMR Characterization of compound **9** minor isomer (CDCl_3_, 22 mM, 293 K, 300 MHz)

| ***Residue*** | ***NH*** | ***α-H*** | ***β-H*** | ***Other*** | ***Noesy*** | ***Δδ/ΔT*** |
| --- | --- | --- | --- | --- | --- | --- |
| tBuCOO |  | - | - | 1.40 (s) | - | - |
| Phe | 5.32-5.24 (m, overl.) | 4.57-4.54 (m, overlap) | 3.07-2.93 (m) | 7.28-7.23 (m, aromatic) |  | Overl. |
| Scaffold |  |  | H3a: 5.28-5.22 (m, overl.)  H4: 3.63-3.61 (m, overl.)  4.01-3.98 (m, overl.)  H6: 4.00-3.87 (m, overl.) 3.56-3.49 (m, overl.)  H6a: 4.06-4.00 (m) | |  | - |
| Leu | 7.07 m overlap | 4.36-4.31 m overlap | 1.86-1.57 (m overl.) | 0.91 (m, overl.) |  | overlapped |
| Val | 6.70 (d, *J*= 8.5) | 4.36-4.31 m overlap | 2.17-2.04 (m, overl.) | 0.91 overl. |  | 2 |
| NH_2_ | 6.08 bs 5.99 bs |  |  |  |  | >5 |

*Compound* ***10***

**Tables S7**. NMR Characterization of compound **10** (CDCl_3_, 18 mM, 293 K, 300 MHz)

| ***Residue*** | ***NH*** | ***α-H*** | ***β-H*** | ***Other*** | ***Noesy (distance)*** | ***Δδ/ΔT*** |
| --- | --- | --- | --- | --- | --- | --- |
| tBuCOO |  | - | - | 1.35 (s) | - | - |
| Phe | 5.63 (bs) | 4.33-4.25 (m, overl.) | 2.09-3.18 (m),  2.70-2.93 (m) | 7.30-7.21 (m, aromatic) | NH-NHGly (2.96 Å) | >5 |
| Gly | 7.20 overl. | 4.52-4.47 (m),  3.84-3.80 (m) | - | - | NH-NHPhe (2.96 Å)  Hα-NHVal  (3.03 Å)  Hα-HβLeu  (2.73 Å)  Hα-NHVal  Hα-MeLeu | >5 |
| Scaffold | - | - | H3a: 5.38-5.33 (m)  H6a: 4.28-4.22 (m, overl.)  H4: 4.18-4.15, 3.53-3.47 (m, overl.)  H6: 3.84-3.76, 3.56-3.47 (m, overl.) | |  |  |
| Leu | 7.47 (d, *J*=6.9 Hz) | 4.24-4.22 (m, overl.) | 1.73-1.66 (m) | 1.73-1.66 (m, overl.), 0.94 (d, overl.)  0.97 (d, overl.) | NH-NHVal  (2.68 Å)  NH-HαGly  Hβ-HαGly  (2.73 Å) | 3 |
| Val | 6.95 (d, *J*=8.5 Hz) | 4.30-4.27 (m) | 2.12-1.96 (m) | 0.87 (d, overl.),  0.93 (d, overl.) | NH-NHLeu  (2.68 Å)  NH-HαGly  (3.03 Å) | 2 |
| NH_2_ | 6.72 (bs), 6.42 (bs) | - | - | - |  | >5  4 |

**^1^H-NMR and ^13^C-NMR spectra of compounds 1, 4-10**


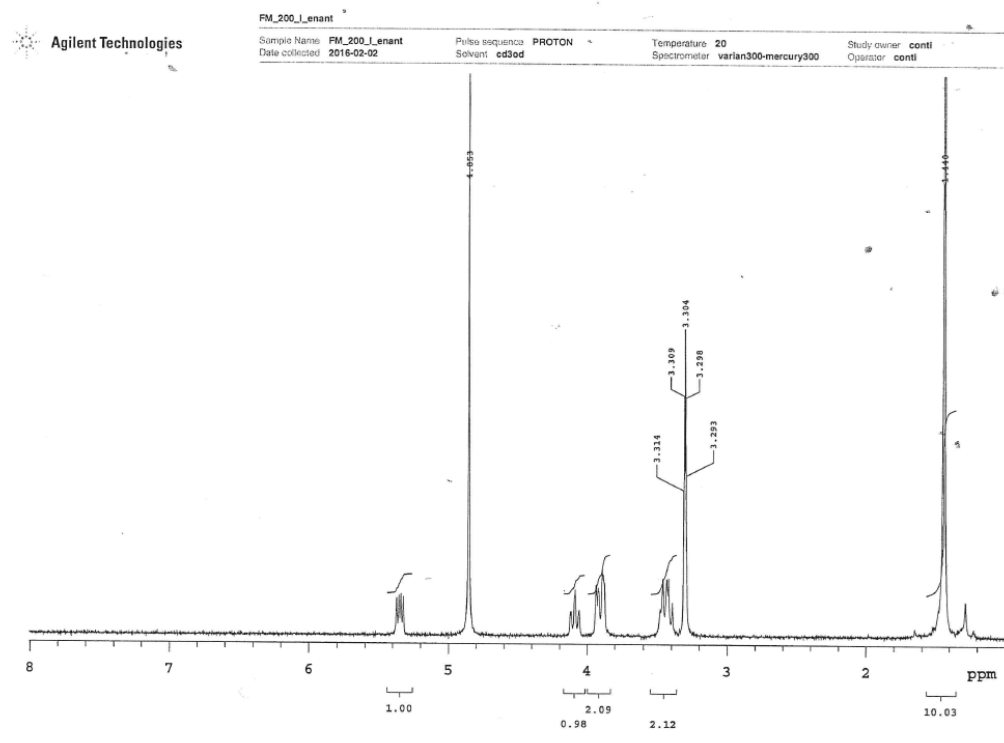


**Figure S1**. ^1^H NMR of Compound **1** in CD_3_OD


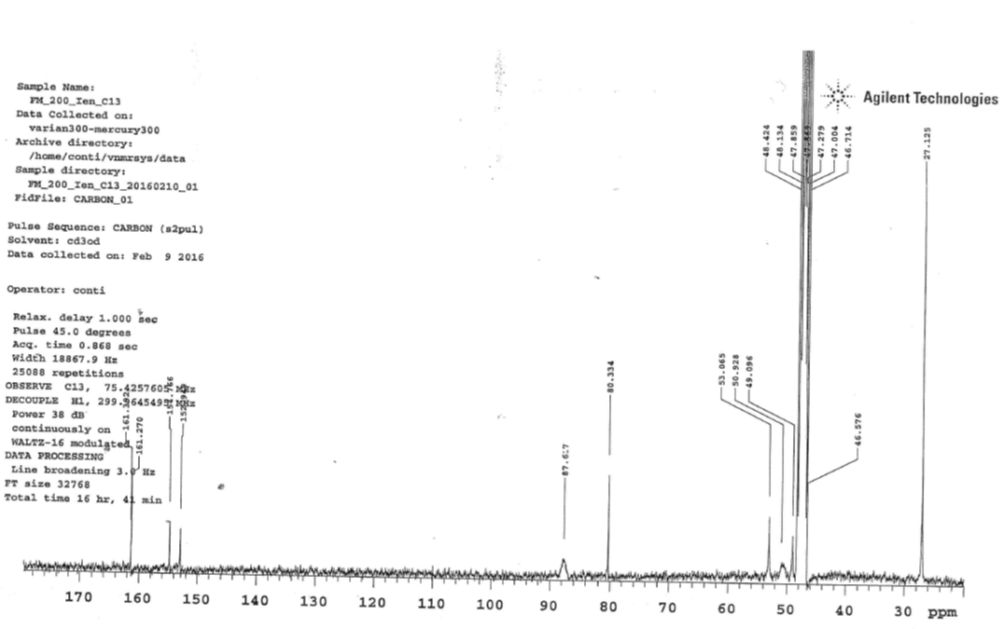


**Figure S2**. ZGDC ^13^C NMR of Compound **1** in CD_3_OD


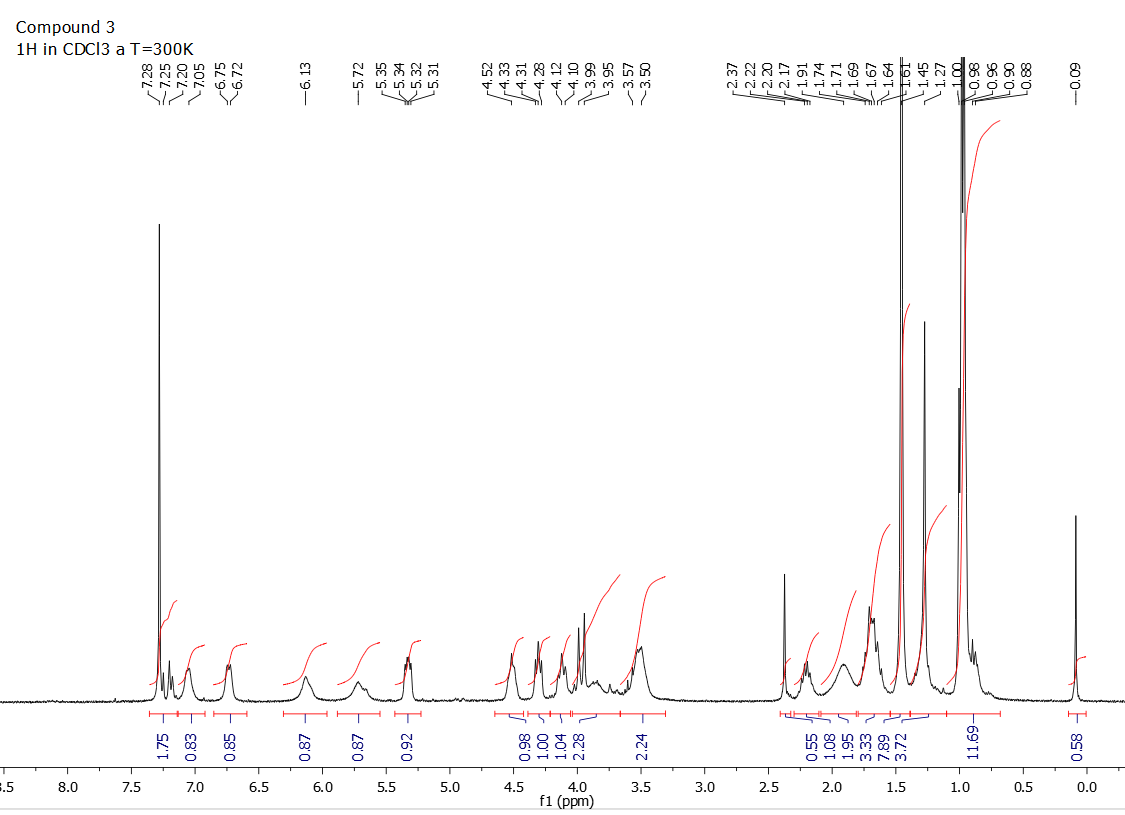


**Figure S3**. ^1^H NMR of Compound **4** in CDCl_3_


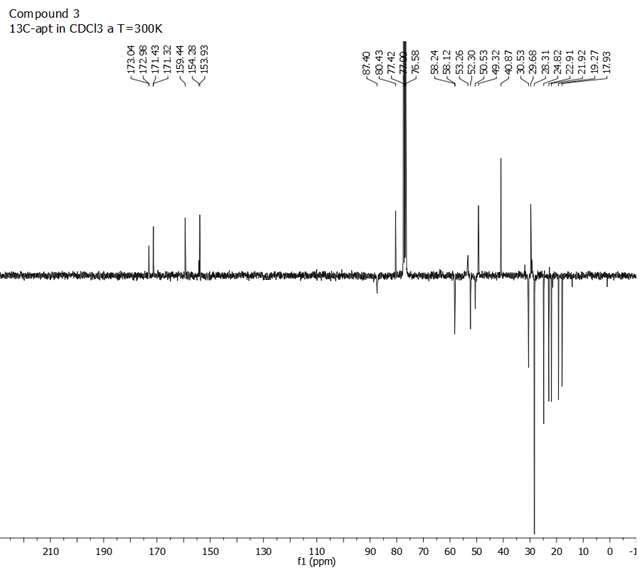


**Figure S4**. APT ^13^C NMR of Compound **4** in CDCl_3_


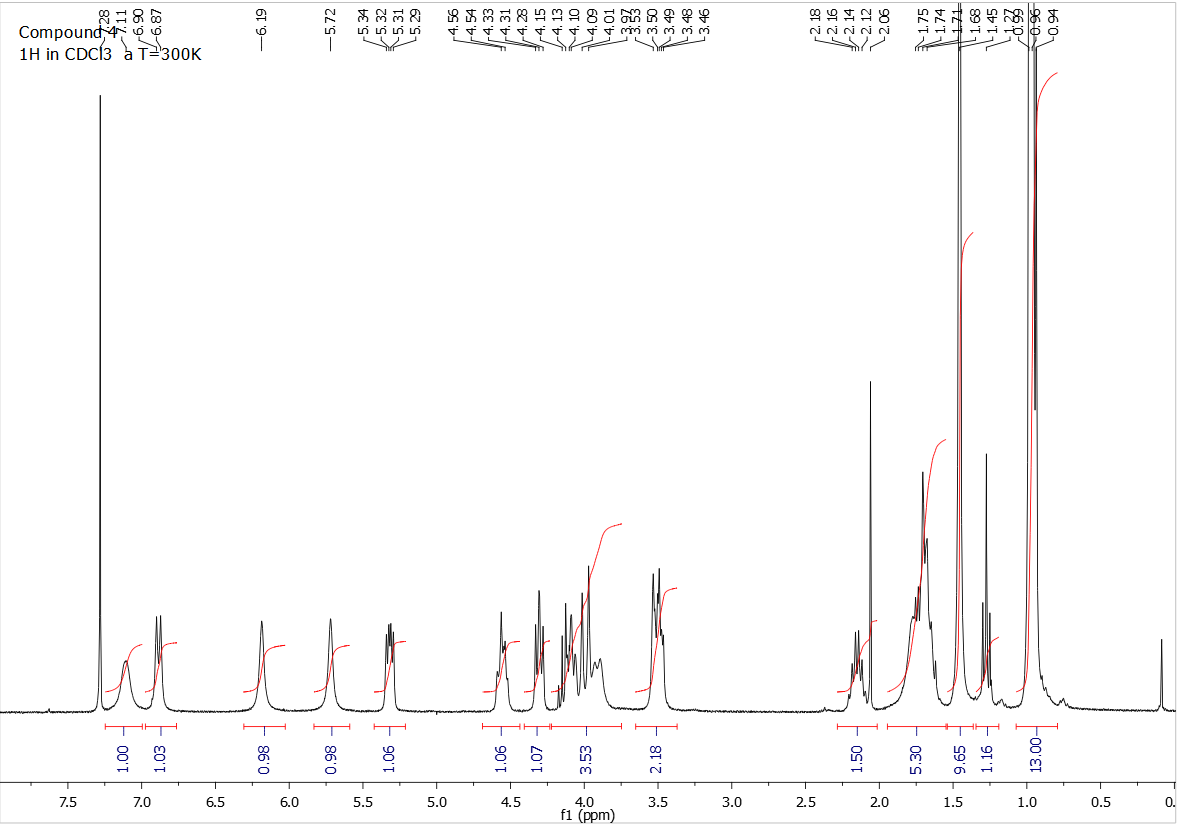


**Figure S5**. ^1^H NMR of Compound **5** in CDCl_3_


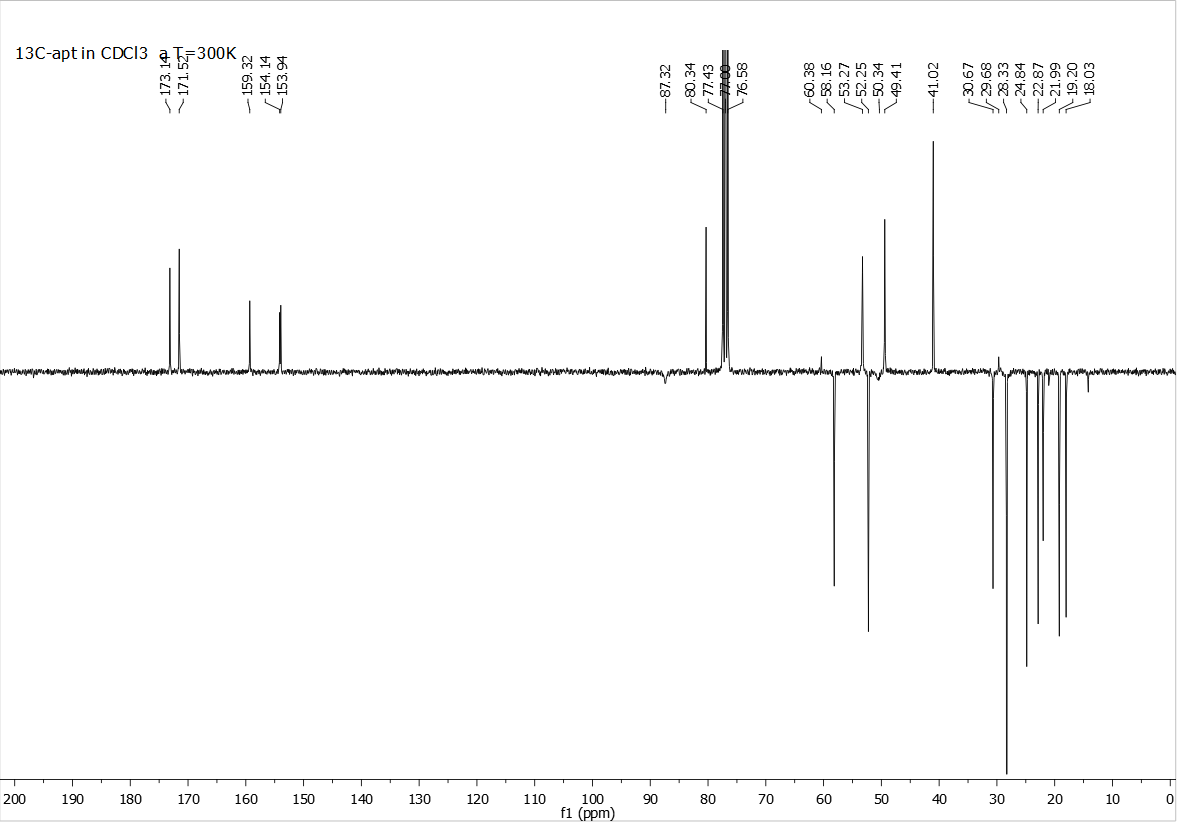


**Figure S6**. APT ^13^C NMR of Compound **5** in CDCl_3_

**
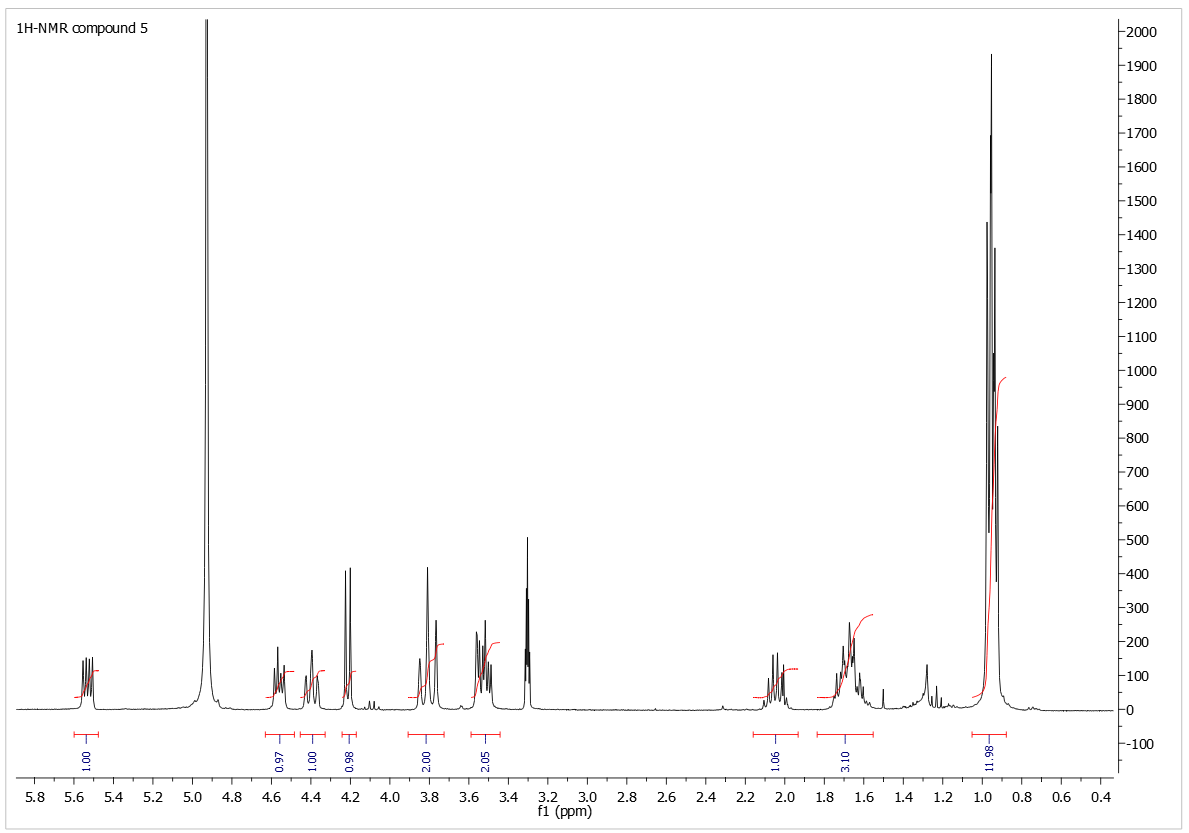
**

**Figure S7**. ^1^H NMR of Compound **6** in CD_3_OD

**
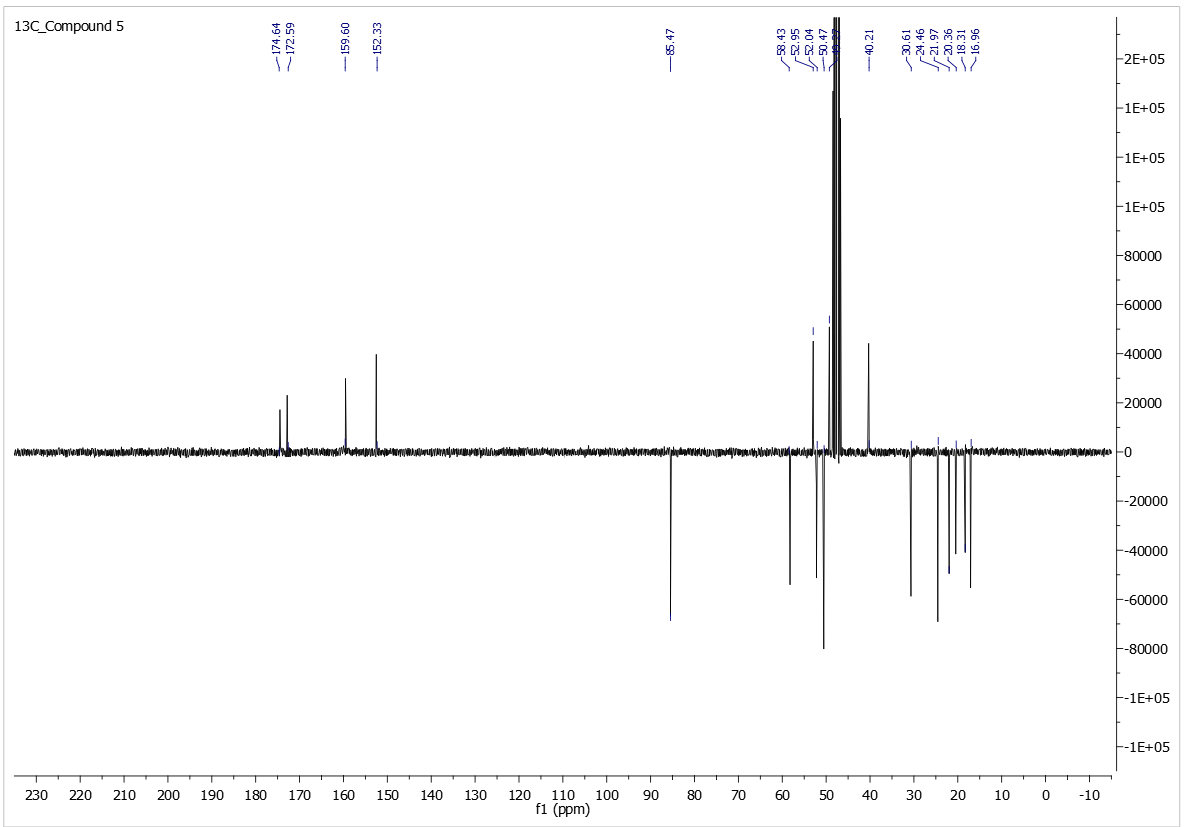
**

**Figure S8**. APT ^13^C NMR of Compound **6** in CD_3_OD

**
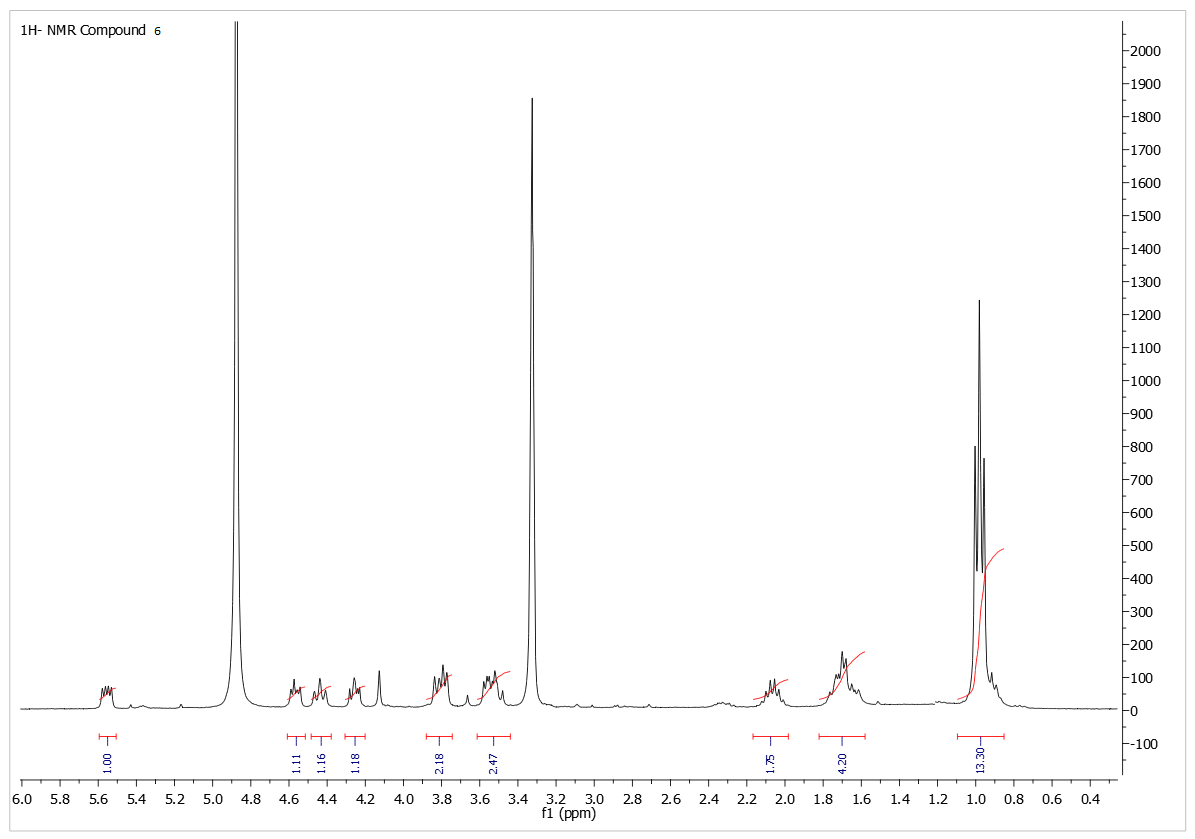
**

**Figure S9**. ^1^H NMR of Compound **7** in CD_3_OD


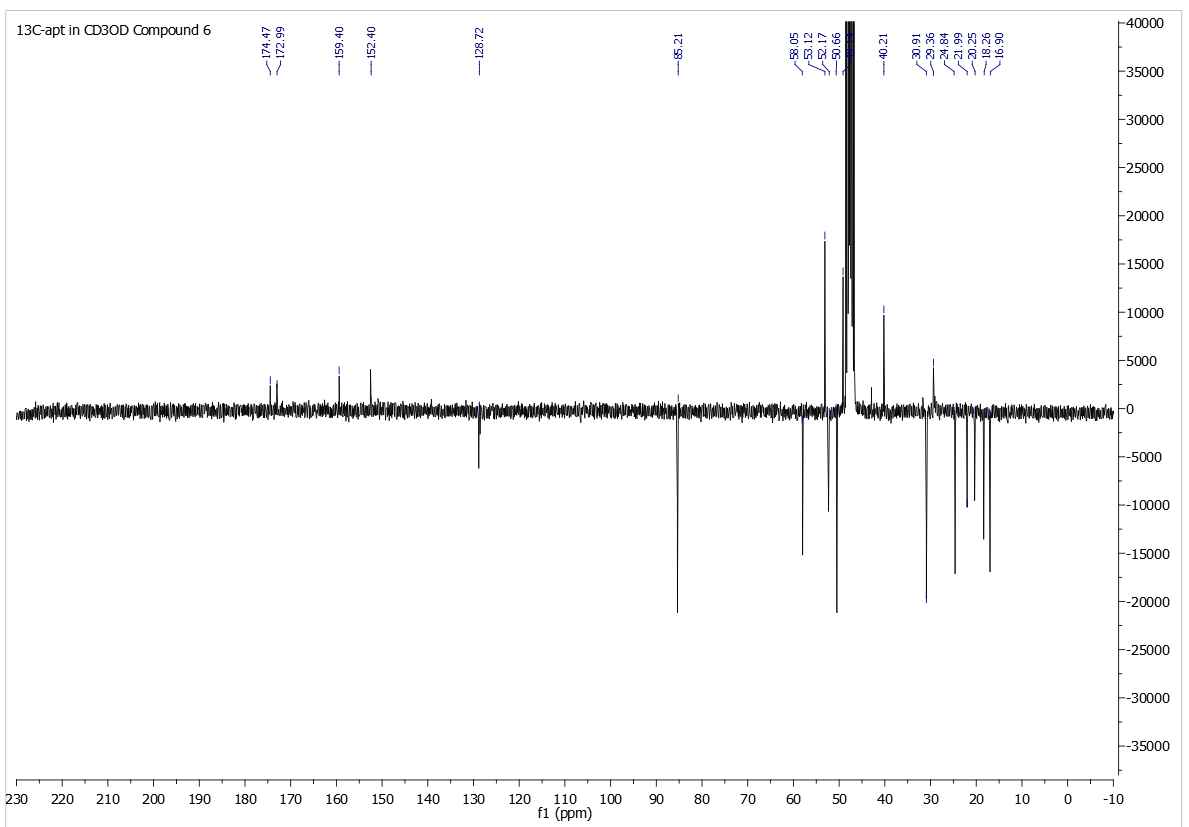


**Figure S10**. APT ^13^C NMR of Compound **7** in CD_3_OD

**
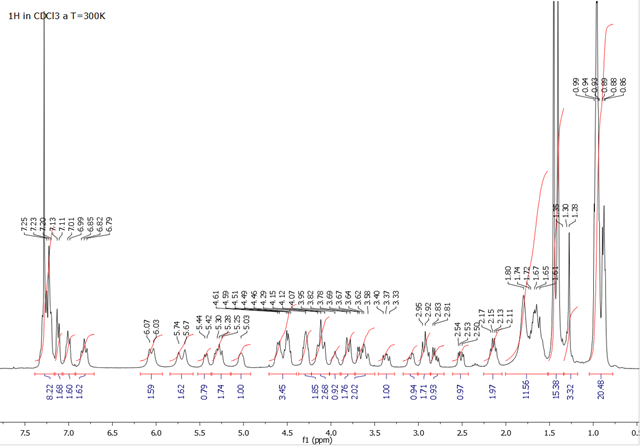
**

**Figure S11**. ^1^H NMR of Compound **8** in CDCl_3_

**
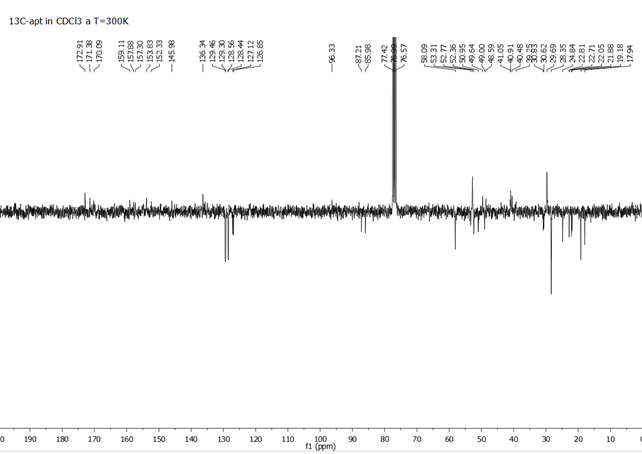
**

**Figure S12**. APT ^13^C NMR of Compound **8** in CDCl_3_

**
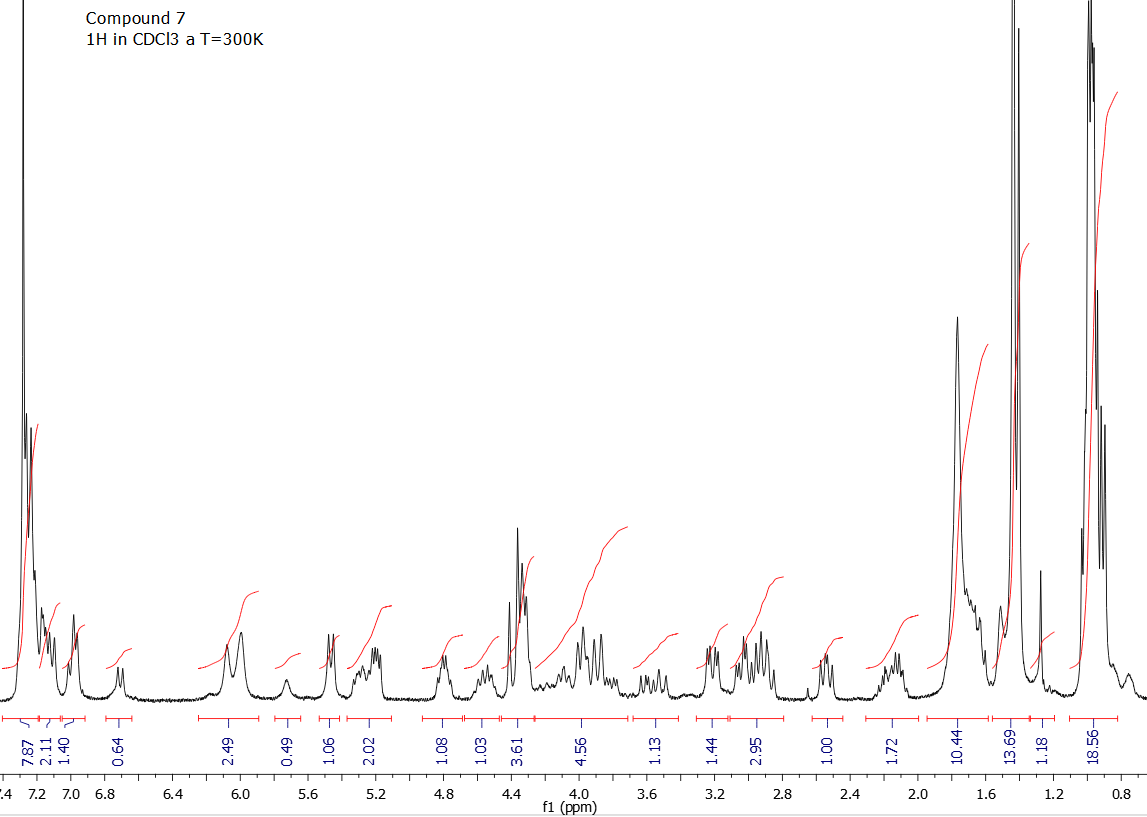
**

**Figure S13**. ^1^H NMR of Compound **9** in CDCl_3_

**
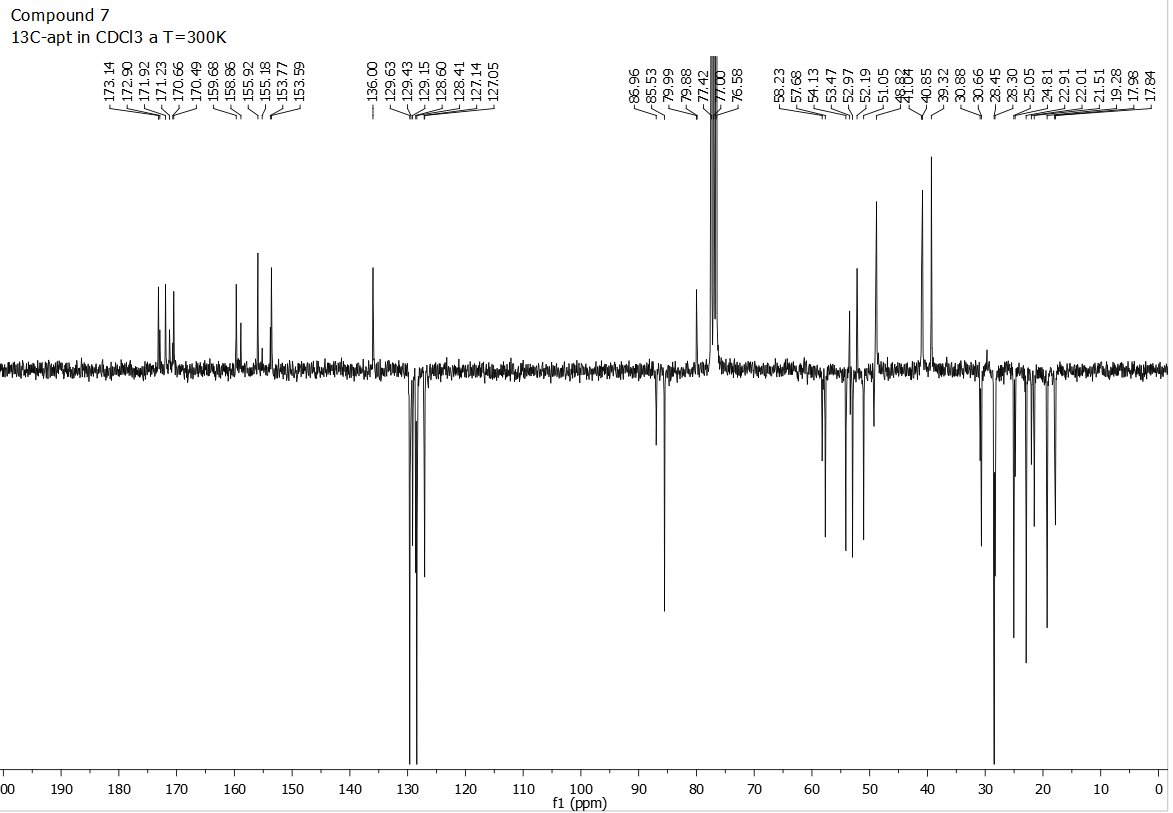
**

**Figure S14**. APT ^13^C NMR of Compound **9** in CDCl_3_


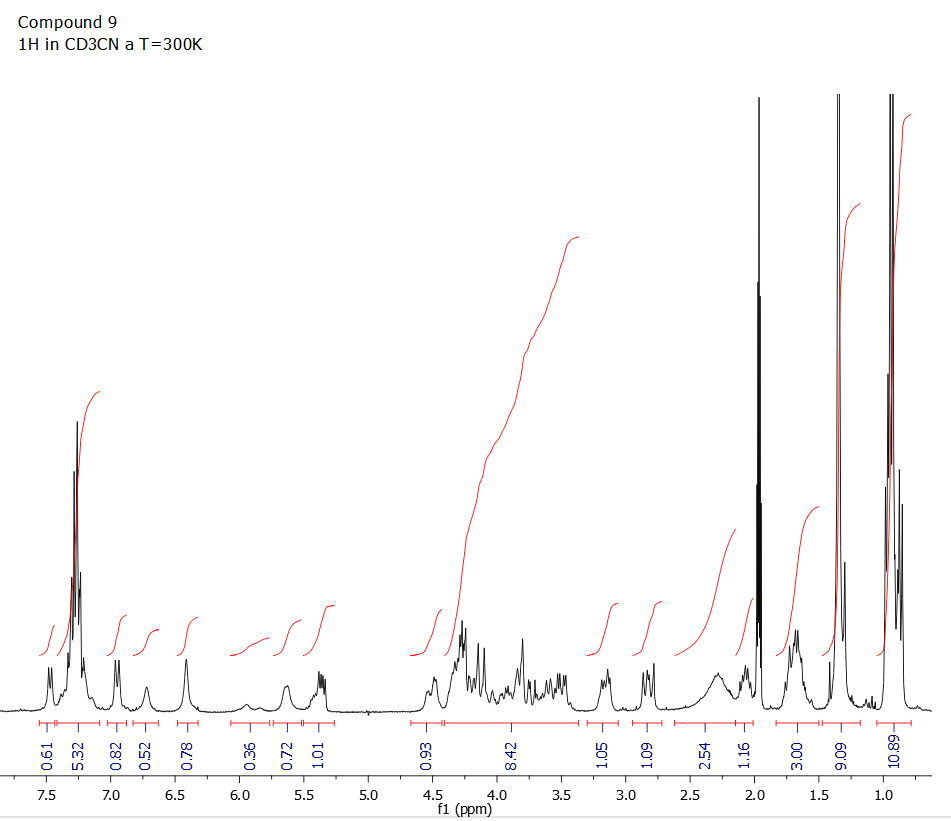


**Figure S15**. ^1^H NMR of Compound **10** in CDCl_3_

**
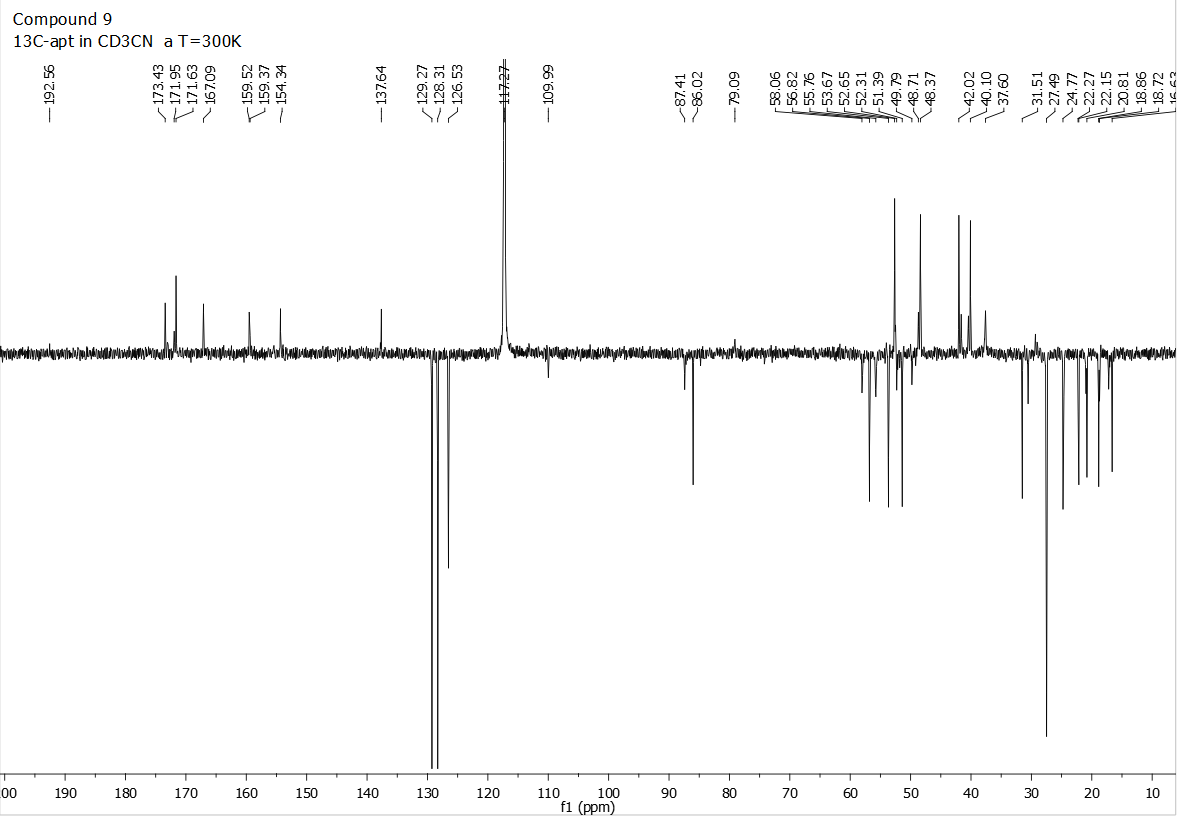
**

**Figure S16**. APT ^13^C NMR of Compound **10** in CDCl_3_


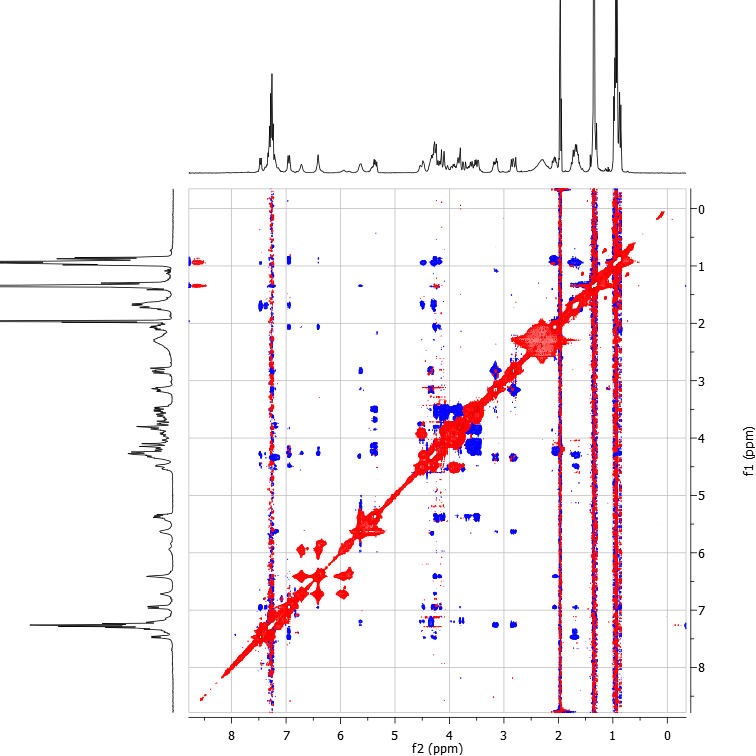


**Figure S17**. Noesy Spectrum of Compound **10** (tmix= 300ms, T= 300K)

**
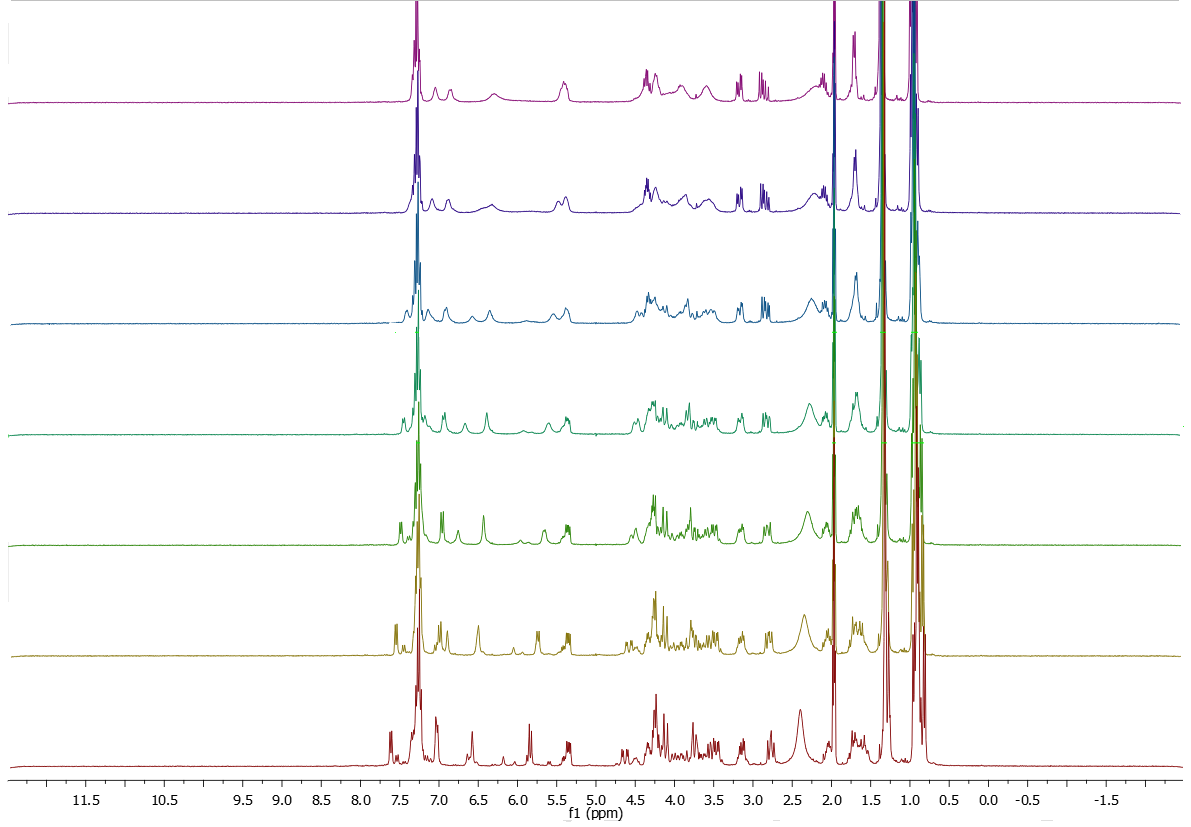
**

**Figure S18**. ^1^H NMR of compound **10** at variable temperatures.


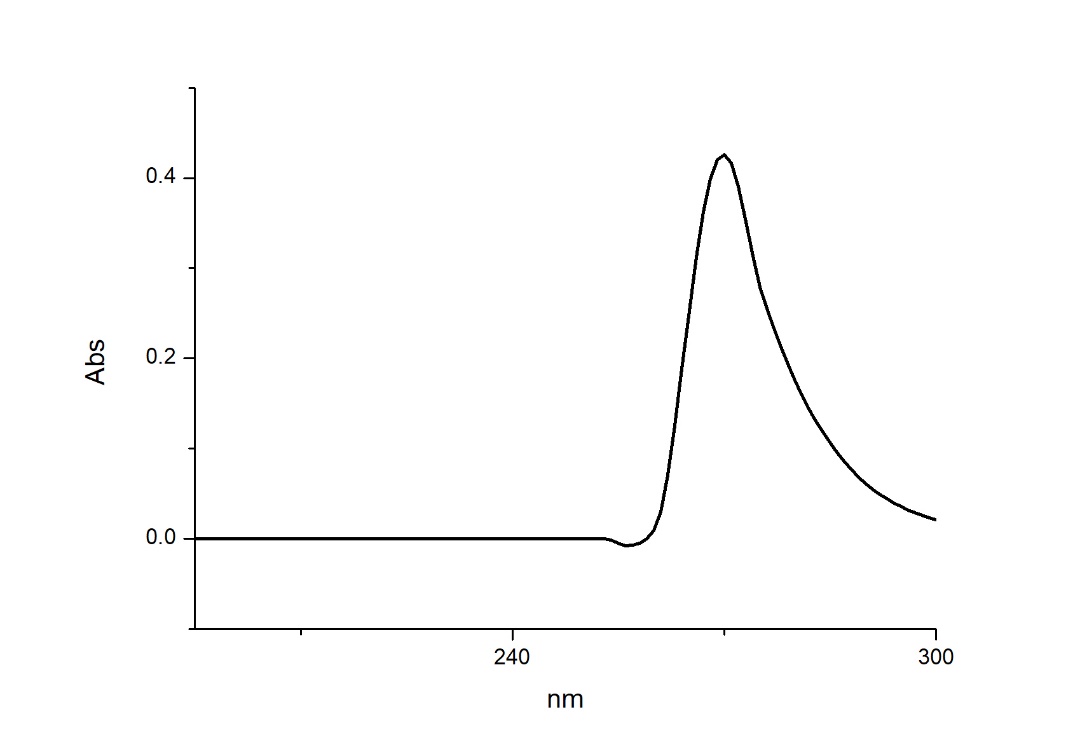


**Figure S19**. UV spectrum of Compound **10** (200 μM in MeOH)

**
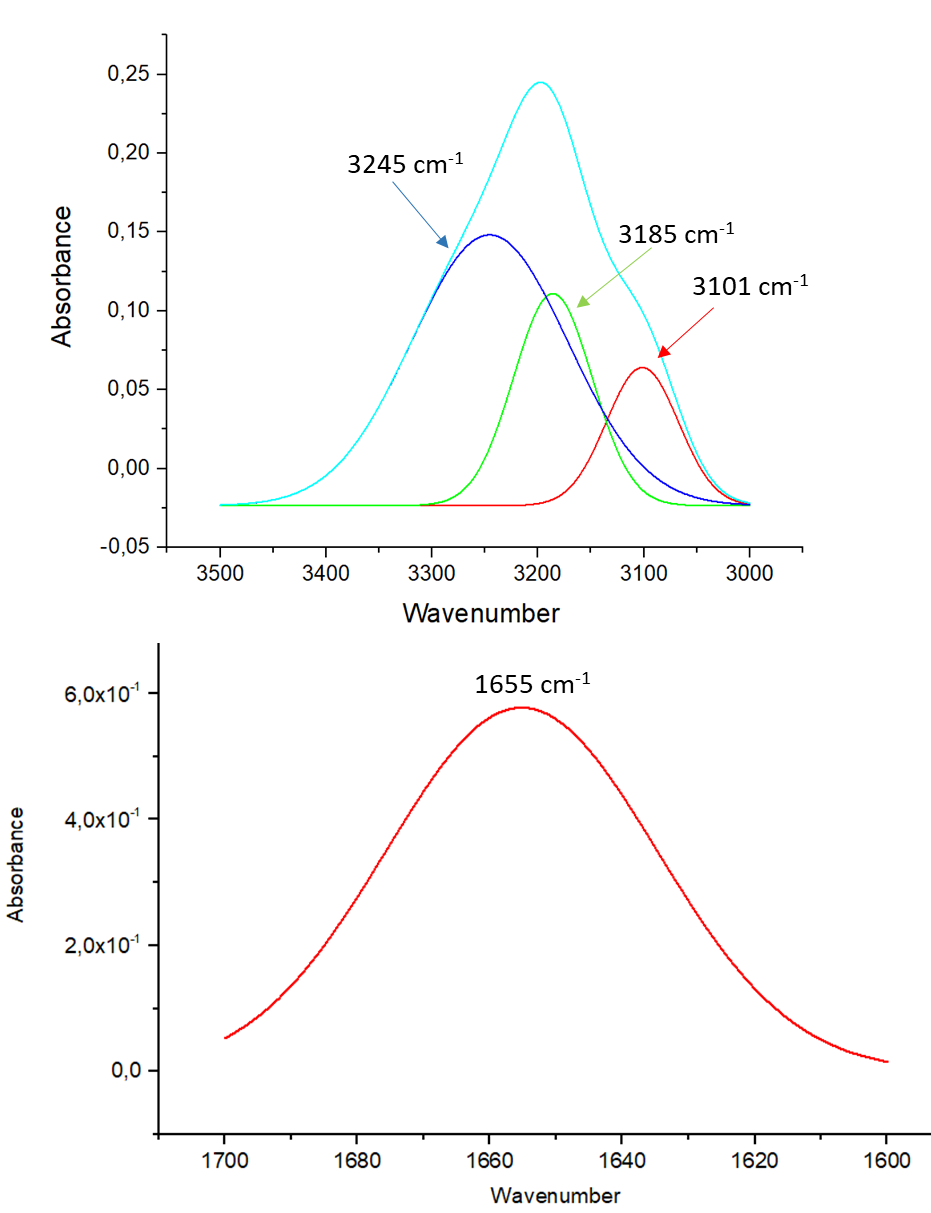
**

**Figure S20.** Deconvoluted FT-IR spectra of NH-stretching (top) and amide I (bottom) regions

**Molecular Modelling:**


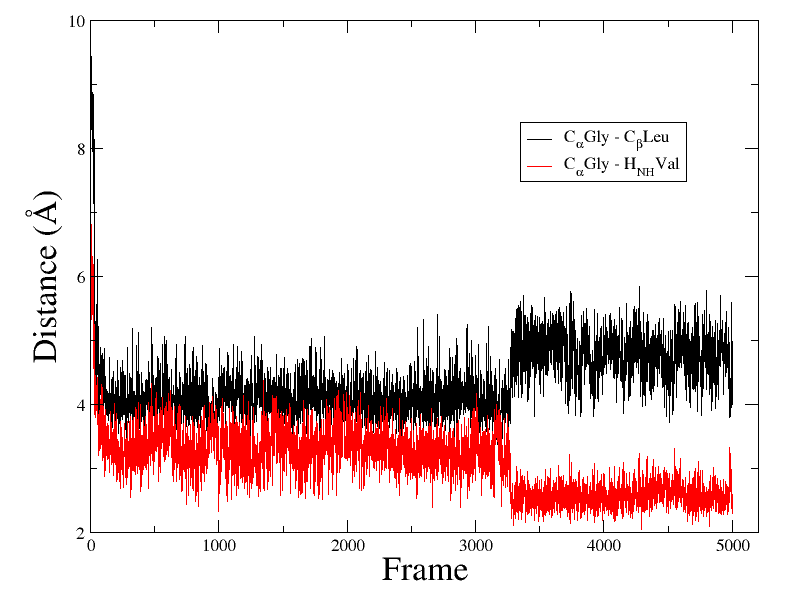


**Figure S21.** Two of the four monitored distances on compound **10**


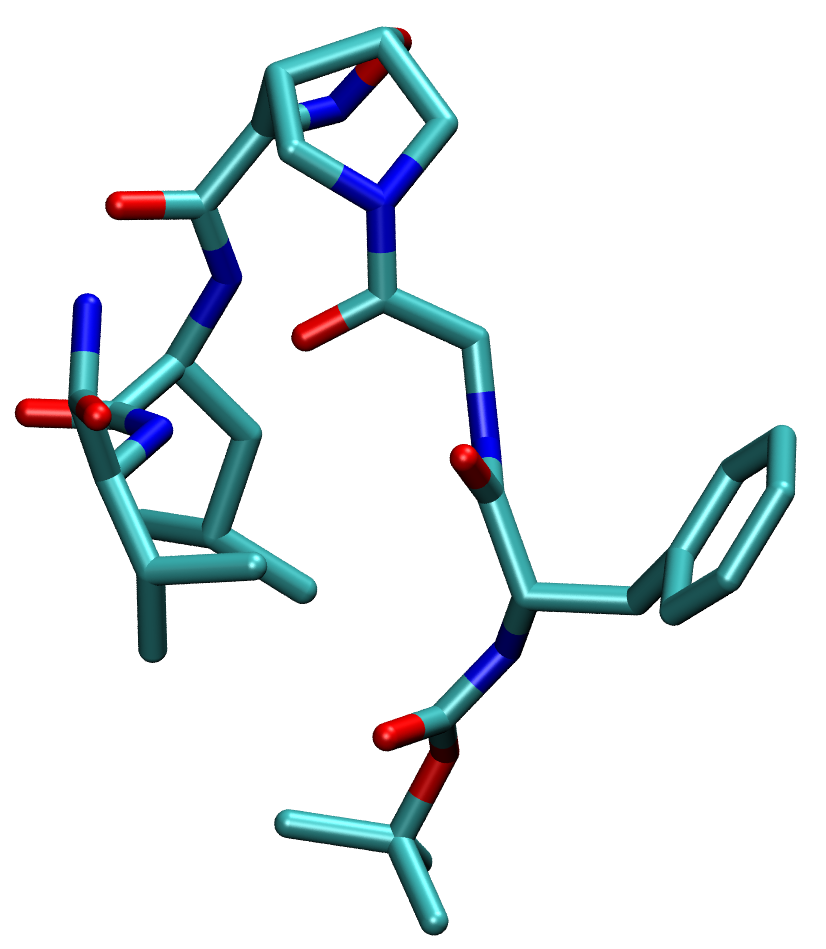


**Figure S22** Compound *trans-***10** during the restained MD.

**Table S8**. Each NOESY value is the center of the restraints windows which has a flat region of 0.85 Å width.

| Atom | Atom | NOESY Value (Å) | *Restaint Average (Cis)* | *Restraint Average (Trans)* |
| --- | --- | --- | --- | --- |
| C_α_Gly | C_β_Leu | 2.73 | 4.35 | 4.80 |
| C_α_Gly | H_NH_Val | 3.03 | 3.08 | 2.63 |
| H_NH_Leu | H_NH_Val | 2.68 | 3.23 | 3.05 |
| H_NH_Phe | H_NH_Gly | 2.96 | 3.94 | 4.22 |


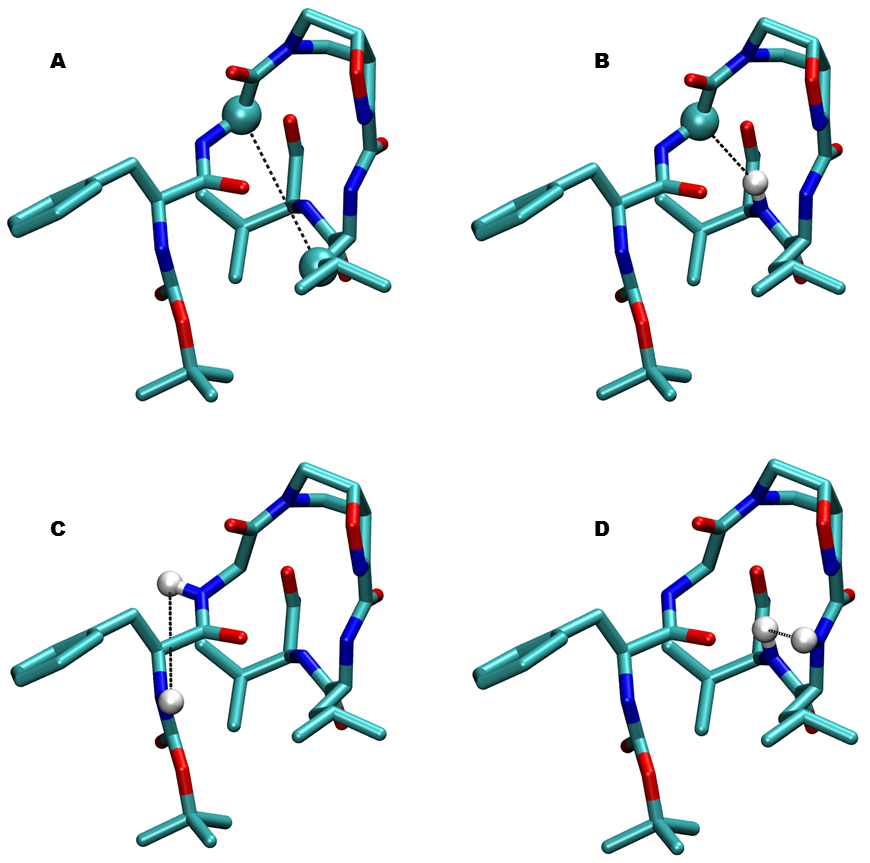


**Figure S23** Interatomic distances restrained during the simulation on compound **10**. C_α_Gly - C_β_Leu (A). C_α_Gly - H_NH_Val (B), H_NH_Phe - H_NH_Gly (C). H_NH_Leu - H_NH_Val (D). Hydrogens are omitted for clarity.

**Table S9.** Dihedral angles and distances

| Φ _i+3_ | ψ _i+3_ | Cα_i_- Cα_i+3_ (Å) | Cα_i_- Cα_i+4_ (Å) | O_i_-N _i+4_ (Å) |
| --- | --- | --- | --- | --- |
| -39.2 | -44.4 | 4.96 | 5.44 | 3.37 |
|  |  |  |  |  |
